# Silibinin-Loaded Liposomes: The Influence of Modifications on Physicochemical Characteristics, Stability, and Bioactivity Associated with Dermal Application

**DOI:** 10.3390/pharmaceutics16111476

**Published:** 2024-11-19

**Authors:** Amjed Abdullah Karkad, Andrea Pirković, Milena Milošević, Bojan Stojadinović, Katarina Šavikin, Aleksandar Marinković, Aleksandra A. Jovanović

**Affiliations:** 1 Faculty of Technology and Metallurgy, University of Belgrade, 11000 Belgrade, Serbia; marinko@tmf.bg.ac.rs (A.M.); 20214044@estudent.tmf.bg.ac.rs (A.A.K.); 2 Faculty of Medical Technology, Elmergib University, Msallata 7310500, Libya; 3 Institute for the Application of Nuclear Energy INEP, University of Belgrade, 11080 Belgrade, Serbia; andrea.pirkovic@inep.co.rs; 4 Institute of Chemistry, Technology and Metallurgy—National Institute of the Republic of Serbia, University of Belgrade, 11000 Belgrade, Serbia; milena.milosevic@ihtm.bg.ac.rs; 5 Institute of Physics Belgrade, University of Belgrade, 11080 Belgrade, Serbia; bojans@ipb.ac.rs; 6 Institute for Medicinal Plants Research “Dr Josif Pančić”, 11000 Belgrade, Serbia; ksavikin@mocbilja.rs

**Keywords:** anti-inflammatory activity, antioxidant activity, encapsulation, liposomes, silibinin, skin, stability

## Abstract

Background/Objectives: The aims of the presented study were the development of four types of silibinin-loaded liposomes (multilamellar liposomes—MLVs, sonicated small unilamellar liposomes—SUVs, UV-irradiated liposomes, and lyophilized liposomes) and their physicochemical characterization and biological potential related to skin health benefits. Methods: The characterization was performed via the determination of the encapsulation efficiency (EE), particle size, polydispersity index, zeta potential, conductivity, mobility, storage stability, density, surface tension, viscosity, FT-IR, and Raman spectra. In addition, cytotoxicity on the keratinocytes and antioxidant and anti-inflammatory potential were also determined. Results: UV irradiation significantly changed the rheological and chemical properties of the liposomes and increased their cytotoxic effect. The lyophilization of the liposomes caused significant changes in their EE and physical characteristics, decreased their ABTS and DPPH radical scavenging potential, and increased their potential to reduce the expression of interleukin 1 beta (IL-1β) in cells treated with bacterial lipopolysaccharide. Sonication significantly changed the EE and physical and rheological properties of the liposomes, and slightly increased their cytotoxicity and reduction effect on IL-1β, while the anti-ABTS and anti-DPPH capacity of the liposomes significantly increased. All developed liposomes showed an increasing trend in particle size and a decreasing trend in zeta potential (absolute values) during storage. Conclusions: Silibinin-loaded liposomes (MLVs and lyophilized) showed promising antioxidant activity (toward reactive oxygen species generated in cells) and anti-inflammatory effects (reducing macrophage inhibitory factor expression) on keratinocytes and did not lead to a change in their viability. Future perspectives will focus on wound healing, anti-aging, and other potential of developed liposomes with silibinin in sophisticated cell-based models of skin diseases, wounds, and aging.

## 1. Introduction

Products from plant material are of great interest to researchers, not only as a source of new biologically active ingredients for use in the pharmaceutical and cosmetic industries but also as a valuable addition to the formulations for improving the aesthetic properties of wounds, burns, scars, and skin as a whole [1,2]. Silibinin, the flavonolignan compound, is the major active constituent of silymarin, a group of polyphenols from milk thistle (*Silybum marianum*), which, apart from silibinin, contains isosilybin, silydianin, and silychristand [3]. Silibinin is also found in artichokes (*Cynara scolymus*) [4]. Due to their antioxidant, antimicrobial, anti-inflammatory [3,5], antiviral [6], immunomodulatory [7], and anticancer potential [8], the mentioned polyphenols exhibit plenty of bioactivities that can promote human health and wellbeing. Silibinin was used as a chemo-preventive and therapeutic agent in human lung cancer [9], while studies have reported that it also showed significant chemo-preventive activity in animal models of carcinogenesis, including prostate and skin cancer [4,10]. According to the Song et al. study [11], silibinin can also protect liver cells against toxins, while García-Viñuales et al. [12] suggest that it can inhibit amyloid-beta aggregation by affecting the human islet amyloid polypeptide. The study of Matsumura and Ananthaswamy [13] shows a protective effect of silibinin against ultraviolet B-induced skin injuries. Namely, silibinin protects from photo-carcinogenesis, sunburns, UVB-caused epidermal hyperplasia, and deoxyribonucleic acid damage, and changes cell cycle regulation in favor of maintaining the genetic integrity of the skin cells [4,5]. However, its application is quite limited due to its poor water solubility, limited absorption, and consequently, low bioavailability [14]. Thus, silibinin requires encapsulation for further application with the aim of its enhanced bioavailability.

The encapsulation of biologically active components represents a technique that has been widely used in the food, pharmaceutical, and cosmetic industries to strengthen and supplement formulations by enhancing stability and bioavailability and controlling the delivery of active compounds [3,15,16,17]. Liposomes, as spherical micro- or nanoparticles formed by one or multiple phospholipid layers, are widely used as carriers for delivering drugs, antioxidants, proteins, enzymes, polyphenols, vitamins, flavors, and aromas, due to their non-toxicity, biodegradability, and ability to encapsulate hydrophilic, amphiphilic, and lipophilic compounds [16,18,19,20,21]. Liposomes can provide a controlled release of biologically active compounds, as well as aiding their protection from modification and degradation caused by light, oxygen, UV irradiation, free radicals, enzymes, changes in pH values, etc. [3,16]. Additionally, several studies have shown that liposomes provide a higher bioavailability of various compounds, including drugs, proteins, nutraceuticals, and polyphenols, due to their lipid composition being similar to that of epithelial cells [22,23,24,25]. The liposomal particles can be formulated using the common thin film hydration procedure, micro-emulsification, membrane extrusion, proliposome method, ether or ethanol injection, a reverse phase evaporation method, etc. [16,19,26]. Among all the previously mentioned techniques, the proliposome technology may be suitable for large-scale production [19]. Additionally, the sonication and cavitation effects can increase the dispersion of lipid molecules, consequently, reducing the particle size of the lipid droplets; thus, the mentioned process is widely employed for obtaining small unilamellar liposomes [26,27,28]. Liposomal vesicle size displays an important influence on the delivery and penetration of encapsulated compounds through the skin, as well as the efficiency of the applied formulation [29]; thus, that was one of the criteria for choosing modification methods during liposome development. Since UV irradiation is used in the food, pharmaceutical, and cosmetic industries as a sterilization technique, and can enhance the release of active compounds from the liposomes and cause changes in the physicochemical properties of the liposomes, its influence should be investigated as well [30]. Also, the potential application of silibinin-loaded liposomes for the dermal and transdermal delivery of silibinin and, consequently, exposure of the formulation to UV rays from the sun, significantly affected the selection of this specific liposome modification technique. Lyophilization (freeze drying), as a simple and frequently employed procedure for drying thermosensitive components, uses freezing and low pressure with the addition of heat (only to cause the sublimation of ice) and can be applied to liposomal vesicles. Namely, the obtained lyophilized products with active compounds (e.g., polyphenols) are stable over a long period, due to the prevention of hydrolytic and oxidative degradation, which can occur in the surrounding water [26,31,32]. Considering that liposomal formulation can contain between 70 and 95% of the water phase, hydrolytic and oxidative reactions, as well as microbiological contamination (which occurs in an aqueous medium), are frequent causes of products’ degradation and spoilage and their short shelf life. With the aim to improve storage stability and provide prolonged shelf life of the liposomal preparations, freeze drying can be used as a simple preservation procedure. However, the lyophilization process can result in significant modifications of the liposomal particles; thus, its effect should be examined as well. Hence, due to all mentioned above related to lyophilization, this process was selected as one of the methods that could potentially have positive or negative impacts on liposome characteristics and bioactivities.

Therefore, the present study aimed to develop silibinin-loaded liposomes using the proliposome procedure, as well as additional steps for liposome modification (sonication by the ultrasound probe, UV irradiation, or lyophilization): multilamellar vesicles (MLVs), sonicated small unilamellar vesicles (SUVs), UV-irradiated liposomes, and lyophilized liposomes, respectively. The encapsulation efficiency (EE), particle size, polydispersity index (PDI), zeta potential, conductivity, mobility, storage stability, density, surface tension, viscosity, FT-IR (Fourier Transform Infrared) and Raman spectra, cytotoxicity, antioxidant and anti-inflammatory activity of the obtained liposomes were investigated. To the best of our knowledge, the influence of all previously mentioned processes on physicochemical characteristics, stability, and bioactivity associated with the dermal application of silibinin-loaded liposomes was not investigated. Specifically, the influence of UV irradiation (present during the production process and dermal application) and sonication or lyophilization (widely employed in industrial conditions) on the cytotoxic, antioxidant, and anti-inflammatory potential of silibinin-loaded liposomes on keratinocytes, the liposome stability, and fingerprint spectra were examined for the first time in the present study.

## 2. Materials and Methods

### 2.1. Reagents

Distilled water was purified through a Simplicity UV^®^ water purification system (Merck Millipore, Merck KGaA, Darmstadt, Germany). Phospholipon 90G (Ph, phosphatidylcholine from soybean) was from Nattermann Phospholipids (Cologne-Bocklemünd, Germany) and ethanol was from Thermo Fisher Scientific (Loughborough, UK), while silibinin (≥98%, HPLC grade), 2,2′-azino-*bis* (3-ethylbenzothiazoline-6-sulphonic acid) or ABTS, ascorbic acid, and 2,2-diphenyl-1-picrylhydrazyl or DPPH were from Sigma Aldrich (Steinheim, Germany). HaCaT cells (spontaneously immortalized human keratinocytes) were kindly provided by the Institute for Biological Research “Siniša Stanković”, National Institute of the Republic of Serbia, University of Belgrade, Belgrade, Serbia. Bacterial lipopolysaccharide (LPS; *Escherichia coli* 055:B5), phosphate-buffered saline (PBS), sodium dodecyl sulfate (SDS), and MTT reagent (thiazolyl blue tetrazolium bromide, 1 mg/mL) were from Sigma-Aldrich (St. Louis, MA, USA) and DMEM/F12 cell culture medium (1:1 mixture of Dulbecco’s Modified Eagle’s Medium and Ham’s F-12 nutrient mixture) was from Pan-Biotech (Aidenbach, Germany), while 0.5% Tween was from Thermo Fisher Scientific (Waltham, MA, USA). Cells were maintained in RPMI 1640 (GIBCO BRL, Thermo Fisher Scientific, Waltham, MA, USA) supplemented with 10% fetal bovine serum (FBS) and containing 1% antibiotic–antimycotic mixture (Capricorn Scientific, Ebsdorfergrund, Germany), hereafter referred to as complete medium. Cell-permeable oxidation-sensitive probe-H2DCFDA (2ʹ,7ʹ-dichlorofluorescin diacetate-Calbiochem) was from Merck Millipore, Darmstadt, Germany. Dulbecco’s Modified Eagle Medium/Nutrient Mixture F-12 Ham (DMEM F 12, Biowest, Nuaillé, France), 10% fetal calf serum (FCS, Gibco, Waltham, MA, USA), and 0.25% trypsin-EDTA solution (Institute for Virology, Vaccines, and Serum “Torlak”, Belgrade, Serbia) were also used.

### 2.2. Cell Culture

HaCaT human keratinocytes were kept in 25 cm^2^ tissue culture flasks in a humidified incubator at 37 °C, with 5% CO_2_. They were grown in a complete medium containing DMEM F 12, 10% fetal calf serum, and 1% antibiotic–antimycotic solution. After reaching 70% confluence, the cells were trypsinized (0.25% trypsin-EDTA solution) and seeded in 96-well plates (1.5 × 10^4^ cells/well). They were left to attach to wells for 24 h at 37 °C, 5% CO_2_, before the treatment.

### 2.3. Preparation of the Liposomes

Silibinin-loaded liposomes as multilamellar vesicles (MLVs) were prepared using the proliposome method according to Jovanović et al. [33]. Specifically, a mixture of 10 g of phospholipids, 1 g of silibinin, and 40 mL of ethanol was stirred and heated to 50–60 °C for 15 min. After cooling to 25 °C, 80 mL of ultrapure water was added in small portions. Subsequently, the mixture was stirred for 1 h at 800 rpm. Plain (empty) liposomes (MLVs) were prepared as a control using 2.5 g of phospholipids, 10 mL of ethanol, and 20 mL of ultrapure water. Due to the complete evaporation of ethanol (from loaded and unloaded liposomes), the concentration of phospholipids in a final formulation was 125 mg/mL.

#### 2.3.1. Sonication of the Liposomes

With the aim to reduce vesicle size and obtain SUVs, the samples (MLVs, 20 mL) were sonicated for 15 min (on 30 s-off 10 s) using the ultrasound probe Sonopuls (Bandelin, Berlin, Germany) at 40% amplitude and 25 °C (a flask with the sample was continuously cooled using ice coating during sonication and the temperature was measured and controlled) [28].

#### 2.3.2. UV Irradiation of the Liposomes

The liposomal sample (MLVs, 20 mL) in a thin layer was exposed to UV-C irradiation (253.7 nm) for 15–90 min in uncovered Petri dishes using a laminar flow cabinet (AC2-4G8, ESCo, Singapore) [30,34].

#### 2.3.3. Lyophilization of the Liposomes

The influence of lyophilization on liposomes was investigated as well. Freshly prepared silibinin-loaded liposomes and empty liposomes (MLVs, 10 mL) were centrifuged, the supernatant was discarded, and the pellet was frozen in the freezer at −80 °C for 1 h and freeze dried at −75 °C and pressure of 0.011 mbar for 24 h and at −65 °C and pressure of 0.054 mbar for one additional hour (Alpha 2–4 LSCplus, Christ, Osterode am Harz, Germany). The lyophilized liposomes were then reconstructed with ultrapure water to their original volume before further analysis of encapsulation efficiency, photon correlation spectroscopy, antioxidant methods, and assays in the cell culture. For FTIR and Raman spectroscopy, UV-irradiated liposomes and SUVs with silibinin were lyophilized in the same way, as were empty and loaded MLVs, to obtain appropriate samples for analysis.

### 2.4. Determination of the Encapsulation Efficiency

The EE was determined using an indirect method and calculated by the amount of silibinin in the supernatant, as shown in Equation (1):

EE [%] = (C_i_ − C_sup_)/C_i_ × 100 (1)

where C_i_ is the initial content of silibinin used for the preparation of liposomes and C_sup_ is the content of silibinin determined in the supernatant.

The free silibinin was removed from the liposome dispersions (MLVs, UV-irradiated, and lyophilized samples) by centrifugation at 17,500 rpm and 4 °C for 45 min in Thermo Scientific Sorval WX Ultra series ultracentrifuge (Thermo Fisher Scientific, Waltham, MA, USA). The free silibinin was removed from SUVs’ dispersion using ultracentrifugation at 10,000 rpm and 4 °C for 5 h (Optima L-90K Ultracentrifuge, Beckman Coulter, Brea, CA, USA). The concentration of silibinin in the supernatants was determined spectrophotometrically at 280 nm (UV Spectrophotometer UV-1800, Shimadzu, Kyoto, Japan).

### 2.5. Photon Correlation Spectroscopy and Storage Stability

The mean size, PDI, zeta potential, conductivity, and mobility of liposomal droplets (MLVs, SUVs, UV-irradiated, and lyophilized samples with silibinin and unloaded MLVs) were measured by photon correlation spectroscopy (PCS). Zetasizer Nano Series, Nano ZS (Malvern Instruments Ltd., Malvern, UK) with the measurement range of 0.6 nm to 6 mm used for the measurement of all the above-mentioned parameters. The analyses were performed at 25 °C, and each sample was diluted 200 times with ultrapure water. Each sample was measured three times, and the results obtained were given as the mean value. The conductivity values are presented as the conductivity factor (1 CF = 10 μS/cm).

All previously mentioned parameters of the silibinin-loaded liposomes (MLVs, SUVs, UV-irradiated, and lyophilized samples) were monitored for 60 days of storage at 4 °C. The measurements were repeated on the 1st, 7th, 14th, 21st, 28th, and 60th days using PCS. During the stability study, lyophilized samples were stored in their dried form and reconstituted before every measurement.

### 2.6. Density, Surface Tension, and Viscosity Analyses

The density and surface tension of three types of silibinin-loaded liposomes (MLVs, SUVs, and UV-irradiated samples) were determined using silicon crystal as the immersion body and Wilhelmy plate, respectively, in Force Tensiometer K20 (KRÜSS, Hamburg, Germany). Each sample (20 mL) was examined three times at 25 °C. The viscosity of the same silibinin-loaded liposomes was also examined using Rotavisc lo-vi device equipment with VOL-C-RTD chamber, VOLS-1 adapter, and spindle (IKA, Staufen, Germany). Each sample (6.7 mL) was examined three times at 25 °C.

### 2.7. FT-IR and Raman Spectroscopy

FT-IR spectra of pure Phospholipon, UV-irradiated Phospholipon, silibinin, and lyophilized MLVs, SUVs, and UV-irradiated samples (since the used spectrometer requires the samples without water) were recorded in the wavenumber between 400 and 4000 cm^−1^ using Nicolet™iS™10spectrometer (Thermo Fisher Scientific, Waltham, MA, USA) with Smart iTR™ Attenuated Total Reflectance (ATR), in 20 scans mode, and at a resolution of 4 cm^−1^. The liposomes were exposed to UV irradiation for 15, 30, 45, 60, 75, and 90 min to detect the duration of the irradiation, which caused the chemical changes in the samples. Additionally, deconvolution of the FT-IR spectra, as a means of more powerful detection with the aim to identify changes in bonding, was performed as well.

The micro-Raman spectra of pure Phospholipon, silibinin, and lyophilized MLVs, SUVs, and UV-irradiated liposomes were collected in a backscattering configuration using a Jobin-Yvon T64000 triple spectrometer equipped with a liquid-nitrogen-cooled CCD camera. Raman scattering spectra were recorded in the range of 150–3400 cm^−1^. The spectral resolution was 2 cm^−1^ and accuracy for all measured wavenumbers is ±3 cm^−1^. The argon/krypton ion laser with an emitting line at *λ* = 514.5 nm was used as an excitation source, with the output laser power kept at less than 1 mW to avoid the heating effects and/or sample degradation.

### 2.8. Antioxidant Capacity of the Liposomes

The antioxidant capacity of all prepared liposomal samples was examined using two antioxidant assays, ABTS and DPPH tests. In addition, the antioxidant potential of the liposomes was investigated in the cell line with generated intracellular free radicals (described in Section 2.9.3).

#### 2.8.1. ABTS Assay

The ABTS radical scavenging potential of silibinin-loaded liposomes was determined using the assay described by Zuhair et al. [35] with a slight modification. The mixture of ABTS solution (5 mL of water and 0.019 g of ABTS powder) and potassium persulfate solution (88 µL) was left to react for 24 h at 4 °C. The ABTS^•+^ working solution was diluted using ethanol (an absorbance of ~0.700 at 734 nm). The ABTS^•+^ solution (2 mL) was mixed with the liposomes (20 µL). After 6 min of incubation, the absorbance was measured, and the radical scavenging activity of the extract was calculated using the following equation (Equation (2)):

ΔA = A_0_ − A_x_ (2)

where A_0_ is the absorbance of the ABTS^•+^ solution, while A_x_ is the absorbance of the ABTS^•+^ solution and the liposomes. The scavenging capacity was expressed as IC_50_ (mg of silibinin/mL of liposomal suspension), which represented the concentration required to neutralize 50% of ABTS^•+^ radicals. Ascorbic acid was used as a positive control.

#### 2.8.2. DPPH Assay

The antioxidant capacity of the liposomal samples was measured via hydrogen donating using the stable DPPH^•^ radicals [35]. Various concentrations of the liposomes (200 μL) were mixed with 2 mL of ethanol DPPH^•^ radical solution (an absorbance of ~0.800 at 517 nm). The absorbance was recorded after 20 min of incubation and the percentage of inhibition was calculated using the following equation (Equation (3)):

% inhibition = (A_0_ − A_x_) × 100/A_0_ (3)

where A_0_ is the absorbance of the control and A_x_ is the absorbance of the DPPH^•^ solution and the liposomes. The results were expressed as IC_50_ (mg of silibinin/mL of liposomal suspension), which represented the concentration required to neutralize 50% of DPPH^•^ radicals. Ascorbic acid was used as a positive control.

### 2.9. Assays on Cell Culture

#### 2.9.1. Treatments Preparation

The stock solution of liposomes with silibinin (MLVs, SUVs, UV-irradiated, and lyophilized samples with silibinin and unloaded MLVs) was prepared, at a concentration of 10 mg/mL and kept at 4 °C. For the experiment, final concentrations of each treatment were prepared from the stock solution by dissolving in fresh complete cell medium to reach final concentrations of 0.1, 1, 10, 25, 50, and 100 µg/mL. These concentrations were further used for cell treatments.

#### 2.9.2. Cytotoxicity Evaluation

The HaCaT cells in complete RPMI medium were seeded in 96-well plates at a density of 1.5 × 10^4^ cells/well, in a final volume of 100 µL per well. The medium was exchanged after 24 h, and treatments were added in a total volume of 100 µL/well. Following the incubation with the treatments (empty MLVs and silibinin-loaded liposomes) or solvent (control) at 37 °C for 24 h, an MTT assay was performed. MTT reagent was added (10 µL per well), and the cells were left for 2 h in the dark at 37 °C for the reaction to occur. Further, purple formazan crystals were dissolved with SDS [36]. Finally, the absorbance was measured at 570 nm on a microplate reader (Epoch, BioTek, Shoreline, WA, USA) after the complete solubilization of the crystals. The data were expressed as percentage viability concerning control (100%). Mean values were represented on bars, from three independent experiments performed in triplicate (n = 9).

#### 2.9.3. H2DCFDA Assay (2′,7′-Dichlorofluorescin Diacetate)

HaCaT cells were left overnight to attach to the wells and kept in a humified incubator at 5% CO_2_ and 37 °C. The next day, the medium was exchanged and silibinin-loaded liposomes (MLVs, SUVs, UV-irradiated, and lyophilized samples) or empty MLVs at final concentrations (0.1, 1, 10, 25, 50, and 100 µg/mL) in complete medium were added to the cells (100 μL per well). After 24 h, treatments were removed, and cells were rinsed with PBS. Next, the assay was performed in line with the manufacturer’s instructions [37]. Using PBS as the diluent, 5 μM of the cell-permeable oxidation-sensitive probe, H2DCFDA was added to the cells and left for 45 min in the dark. Next, the cells were washed with PBS and exposed to PBS alone (control) or the 200 μM H_2_O_2_, used as the positive control. After an incubation time of 2 h, and the conversion of non-fluorescent H2DCFDA to the highly fluorescent 2′,7′-dichlorofluorescein (DCF), the generation of intracellular ROS (reactive oxygen species) level in cells was determined by measuring the fluorescence on a fluorescent plate reader (Wallac 1420 multilabel counter Victor 3V, PerkinElmer Life and Analytical Sciences, Boston, MA, USA) at excitation and emission wavelengths of 485 and 535 nm, respectively. Data were expressed as relative fluorescence intensity and the mean value was represented in figures, from three independent experiments performed in triplicate (n = 9).

#### 2.9.4. Determination of Protein Expression Using the CELISA (CELL-BASED ELISA) Method

Analysis of the anti-inflammatory potential of the obtained liposomal samples was performed using cell-based ELISA according to the previously described method [38]. Namely, HaCaT cells were seeded in 96-well plates at a density of 2 × 10^5^ cells per well and grown for 24 h at 37 °C and 5% CO_2_. The following day, the medium was replaced with treatments containing MLVs, SUVs, UV-irradiated, and lyophilized samples or empty MLVs at a final concentration of 10 µg/mL in a complete medium and incubated for 24 h with the cells. At the end of the treatment, the medium was removed, and the cells were exposed to 2.5 µg/mL of LPS in a complete medium for 4 h at 37 °C and 5% CO_2_. Afterwards, cells were washed twice with PBS and the plate was dried. After drying, the cells were fixed with ice-cold acetone-methanol (1:1) for 10 min. Next, endogenous peroxidases were blocked by adding 0.3% H_2_O_2_, 100 μL per well for 30 min in the dark. Then, the wells were washed with PBS and blocked with the addition of 1% BSA in PBS for 30 min at 37 °C. After blocking, 50 μL of each primary antibody (PA5-27238, source: rabbit, 1:500, Invitrogen, Waltham, MA, USA) for interleukin 1 beta (IL-1β), macrophage inhibitory factor (MIF), or cyclooxygenase-2 (COX-2) was added in PBS with 1% BSA to the wells and incubated 2 h at room temperature. Following the incubation with antibodies, the plate was washed three times with PBS containing 0.5% Tween, and a secondary antibody (1:2000, anti-rabbit IgG, HRP-linked Antibody 7074P2, Cell Signaling Technology, Danvers, MA, USA; or 1:2000, anti-mouse IgG, HRP-linked Antibody 7076S, Cell Signaling Technology, Danvers, MA, USA) in PBS with 1% BSA was added to the wells and incubation lasted 2 h at room temperature. Finally, the plate was washed three times with PBS, 50 μL of substrate was added to each well, and color development was monitored. When the color developed, 50 μL of the stop reagent was added and the plate was read at 450 nm wavelength on a plate reader (ELx800, BioTek, Shoreline, WA, USA).

### 2.10. Statistical Analysis

All measurements and analyses were performed in triplicate and statistical analyses were carried out using the statistical software STATISTICA 7.0. The statistical significance was determined using analysis of variance (one-way ANOVA), followed by Duncan’s *post hoc* test. The data in the table and graphs are presented as mean value ± standard deviation. The differences were considered statistically significant at *p* < 0.05.

In the cell assays, one-way analysis of variance (ANOVA) with the Tukey *post hoc* test was used to assess differences in treatments *versus* control after data were tested for normality. All results are expressed as mean ± standard error of the mean (mean ± SEM). GraphPad Prism 6.0 (GraphPad Software, Inc., La Jolla, CA, USA) was used for statistical analysis, where *p* < 0.05 was considered significant.

## 3. Results

The first step of the present research was the formulation of silibinin-loaded liposomes and the investigation of the influence of sonication, UV irradiation, and lyophilization on liposome physicochemical properties, including the EE, particle size, PDI, zeta potential, conductivity, mobility, and storage stability. In the case of liquid samples, density, surface tension, and viscosity were also measured. The second step was the analysis of FT-IR and Raman spectra. The third step was the examination of the biological activity of all developed silibinin-loaded liposomes, including their antioxidant, cytotoxic, and anti-inflammatory potential.

### 3.1. Encapsulation Efficiency in the Silibinin-Loaded Liposomes

Regarding the fact that the efficiency of the encapsulation process, i.e., the amount of the encapsulated target compounds, represents one of the essential parameters, the EE of silibinin in four prepared liposomal systems is shown in Table 1.

The EE of silibinin in MLVs amounted to 89.7 ± 1.4% and UV irradiation did not significantly influence the mentioned parameter (88.1 ± 1.2%) (Table 1). SUVs with silibinin showed a significantly lower value of EE (74.9 ± 1.0%) in comparison to larger particles but a significantly higher value compared to lyophilized liposomes, whose EE was 62.5 ± 1.9% (Table 1).

### 3.2. The Particle Size, PDI, Zeta Potential, Conductivity, and Mobility of the Silibinin-Loaded Liposomes

Since the average size of liposomal particles represents an essential and relevant parameter for liposome biodistribution and the release of the encapsulated compounds [20], the measurement of the mentioned variable was performed (Table 1). The average size of the MLVs was 1675.0 ± 44.3 nm. UV irradiation did not affect the vesicle size of silibinin-loaded liposomes, 1701.5 ± 58.7 nm, while sonication caused a significant decrease in the vesicle size, 277.5 ± 10.0 nm. The size of lyophilized liposomes was 724.9 ± 27.5 nm, showing that the lyophilization process led to a diameter decrease. The PDI values for MLVs, UV-irradiated, and lyophilized samples amounted to ~0.3 (Table 1). The highest PDI value, i.e., a narrow range of particle size distribution, was recorded for the SUVs (0.520 ± 0.059) which can mean the presence of MLVs along with SUVs. The zeta potential of the liposomes was measured as the third physical characteristic (Table 1). The zeta potential is used for the determination of the electrical charge present on the surface of the liposomal membrane and all developed liposomes have negative values of zeta potential, demonstrating that the liposome surfaces were negatively charged. The zeta potential of MLVs and their UV-irradiated parallels did not significantly differ and amounted to −35.5 ± 0.7 and −36.5 ± 0.7 mV, respectively. On the other hand, lyophilization and sonication significantly changed the values of liposome zeta potential (−14.9 ± 0.5 and −21.6 ± 0.1 mV, respectively). The conductivity of the liposomes was determined using PCS as well (Table 1). The conductivity factor of the MLVs, UV-irradiated, lyophilized, and SUVs liposomes with silibinin immediately after the preparation was 0.38 ± 0.01, 1.15 ± 0.02, 2.64 ± 0.30, and 1.24 ± 0.08, respectively. The mobility of all four developed liposomal formulations was determined as the fifth physical property (Table 1). The mobility of the MLVs, UV-irradiated, lyophilized, and SUVs liposomes with silibinin immediately after the formulation was −2.78 ± 0.05, −2.89 ± 0.07, −0.70 ± 0.06, and −1.58 ± 0.04 µmcm/Vs, respectively.

### 3.3. Storage Stability of the Silibinin-Loaded Liposomes

**Figure 1:**
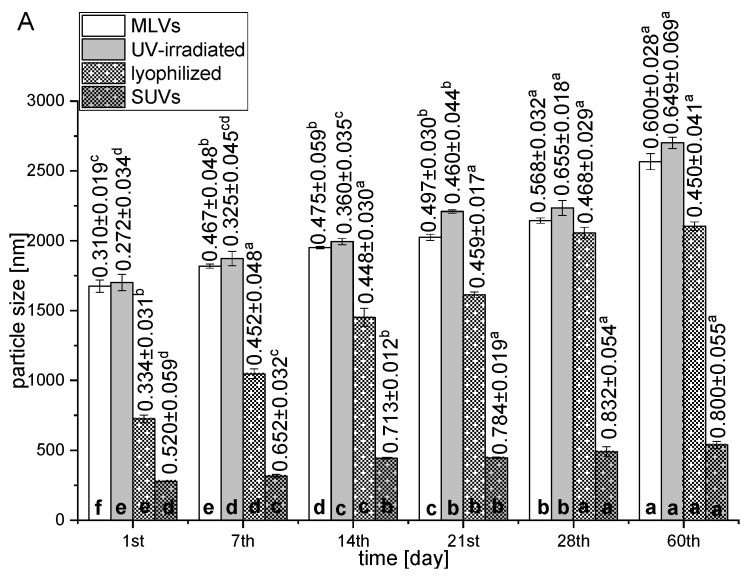

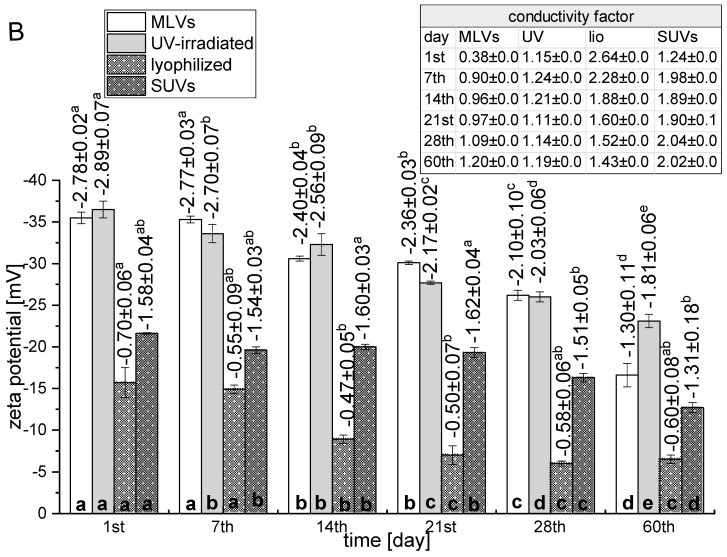
Particle size bars and polydispersity index numbers above bars (**A**) and zeta potential-bars, mobility-numbers above bars [µmcm/Vs], and conductivity-table (**B**) of multilamellar liposomes (MLVs), UV-irradiated liposomes, lyophilized liposomes, and small unilamellar liposomes (SUVs) with encapsulated silibinin monitored for 60 days of their storage at 4 °C; values with the same letter showed no statistically significant difference (*p* > 0.05; n = 3; analysis of variance, Duncan’s *post hoc* test).

One of the most important challenges in the application of liposomal systems within food, functional food, supplements, pharmaceutical, and cosmetic products is their relative physical and chemical instability in water dispersions and under environmental conditions due to their lipid composition, which can lead to unwanted effects, including oxidation and hydrolysis and a reduction in encapsulation efficiency [39,40]. According to the literature data, their physicochemical instability resulted in membrane combination, aggregation, and changes in particle size, rigidity, and membrane compounds, as well as a decrease in encapsulation efficiency [40,41]. Therefore, the storage stability of the developed liposomes was monitored for 60 days at 4 °C, and the results are presented graphically in Figure 1.

The instability of the liposomes can be attributed to the physical collision of the vesicles and membrane fusion, as well as chemical interactions, lipid oxidation, and production of aldehydes. The higher stability of the liposomes at the temperature of 4 °C was due to the permeability and less flexibility of their membranes, consequently causing lower mobility of phospholipids, and the delayed oxidative process of the unsaturated fatty acids and decomposition of the liposomes [42,43]. However, a significant increase in particle size was noticed in all liposomes with encapsulated silibinin during a 60-day storage study at 4 °C (Figure 1A). The initial mean size of liposomal vesicles was 1675.0, 1701.5, 724.9, and 277.8 nm, which, after 60 days of storage at 4 °C, were increased up to 2466.0 nm (by 32%), 2601.0 nm (by 34%), 2104.0 nm (by 65%), and 538.7 nm (by 48%) for MLVs, UV-irradiated, lyophilized, and sonicated forms, respectively. PDI values measured for all liposomal samples during the time (Figure 1A, numbers above bars) show two different behaviors: (i) PDI remained between 0.33 and 0.47 for lyophilized samples, reflecting a slight increase in the heterogeneity and (ii) PDI value significantly increased with storage time for MLVs (from 0.31 to 0.60), UV-irradiated liposomes (from 0.27 to 0.65), and SUVs (from 0.52 to 0.83), indicating less homogeneity and more aggregation. As can be seen in Figure 1B, a significant decrease in the absolute value of zeta potential was observed in all liposomal forms with silibinin for 60 days. The initial zeta potential was −35.5, −36.5, −14.9, and −21.6 mV, which, after the 60-day storage study, were decreased to −16.6 mV (by 53%), −23.1 mV (by 36%), −6.0 mV (by 60%), and −12.7 mV (by 41%) for MLVs, UV-irradiated, lyophilized, and sonicated samples, respectively (Figure 1B). It can be seen that there was a significant increase in the conductivity factor in MLVs and SUVs with silibinin during a 60-day storage study (from 0.38 to 1.20 µmcm/Vs and from 1.24 to 2.02 µmcm/Vs, respectively), while, in the case of the UV-irradiated parallel, the conductivity was not changed (table in Figure 1B). In addition, there was a significant drop in the conductivity factor of the lyophilized sample, from 2.64 to 1.43 µmcm/Vs during storage. The mobility of MLVs and UV-irradiated liposomes significantly decreased during 60 days of storage, whereas in the case of SUVs and lyophilized samples, the decrease in mobility was slower (Figure 1B, numbers above bars). In the MLVs system, mobility decreased from −2.78 to −1.30 µmcm/Vs, while in the UV-irradiated formulation, the mentioned parameter decreased from −2.89 to −1.81 µmcm/Vs. The mobility of SUVs decreased from −1.58 to −1.31 µmcm/Vs, while in the lyophilized liposomal form, it can be seen that there was a drop from −0.70 to 0.47 µmcm/Vs.

### 3.4. The Density, Surface Tension, and Viscosity of the Silibinin-Loaded Liposomes

The physical properties of liquid silibinin-loaded liposomes (density, surface tension, and viscosity) were investigated before and after UV irradiation and sonication. As can be seen in Table 2, the density of MLVs, UV-irradiated liposomes, and SUVs was 0.939 ± 0.005, 0.917 ± 0.004, and 0.916 ± 0.006 g/cm^3^, respectively.

MLVs possessed a significantly higher value of surface tension (28.7 ± 0.1 mN/m) compared with UV-irradiated liposomes and SUVs (27.1 ± 0.2 and 26.5 ± 0.2 mN/m, respectively). The viscosity of all prepared liquid formulations varied in a narrow range, from 3.28 to 3.45 mPa·s.

### 3.5. FT-IR Study

The FT-IR is an easy and versatile analytical tool used for studying the structure and intermolecular interaction in liposome-based systems. Also, the influence of UV irradiation of 2-(oleoyloxy)-3-(stearoyloxy) propyl (2-(trimethylammonio)ethyl) phosphate (phosphatidylcholine), empty liposomes, and silibinin-loaded liposomes was studied using the FT-IR technique. The FT-IR spectra of Phospholipon (phosphatidylcholine, Ph) and empty liposomes (non-loaded liposomes), before and after UV treatment, are shown in Figure S1. The structure of phosphatidylcholine and silibinin A are given in Figure S2. The analysis of the structural changes was based on the inspection of characteristic peaks of intensity change and using deconvolution methodology to separate them into well-resolved structures of functional groups absorption that emerged as a result of the applied treatment.

In the spectrum of Ph broad peak, the range 3600–3000 cm^−1^ is assigned to the O-H stretching vibration (from glycerol and phosphate groups residues) (Figure S1). The small and sharp peak at 3010 cm^−1^ is due to the ethylenic C-H stretching vibration in oleic acid residue in phosphatidylcholine as the main compound of Ph [44]. Additionally, the strong and intensive peaks observed at 2923 cm^−1^ and 2853 cm^−1^ are due to the methyl and methylene groups’ asymmetric and symmetric stretching vibration of the fatty acids residue of phosphatidylcholine. The absorption mode at 1735 cm^−1^, observed in all spectra containing phosphatidylcholine, is assigned to the C=O present in the ester group. The small peak at 1652 cm^−1^ is due to the deformation vibrations of the O-H group and the low contribution of the absorption from the C=C stretching vibration in oleic acid residue. The absorption in the spectral range from 1466 cm^−1^ to 1375 cm^−1^ was assigned to the C-H deformations vibration of the methyl and methylene groups in phosphatidylcholine. The stretching vibration of C-O and C-O-C, as well as the P=O phosphate group in the hydrophilic part of phosphatidylcholine, was observed at 1246 cm^−1^, 1171 cm^−1^, 1086 cm^−1^, and 1063 cm^−1^, respectively. The band at 1062 cm^−1^ was assigned to the C-O and P-O-C stretching band from the phospholipid structure. Also, the peak at 872 cm^−1^ arises from the P-O asymmetric stretching vibration. The stretching vibration of γ(=C-H) in the *cis*-unsaturated double bond of phospholipids appeared in the 730–770 cm^−1^ region, which is overlapped with the O-CO-C bending vibrations that originate from a molecule of phosphatidylcholine (usually observed at a wavenumber of 734 cm^−1^) [45].

**Figure 2:**
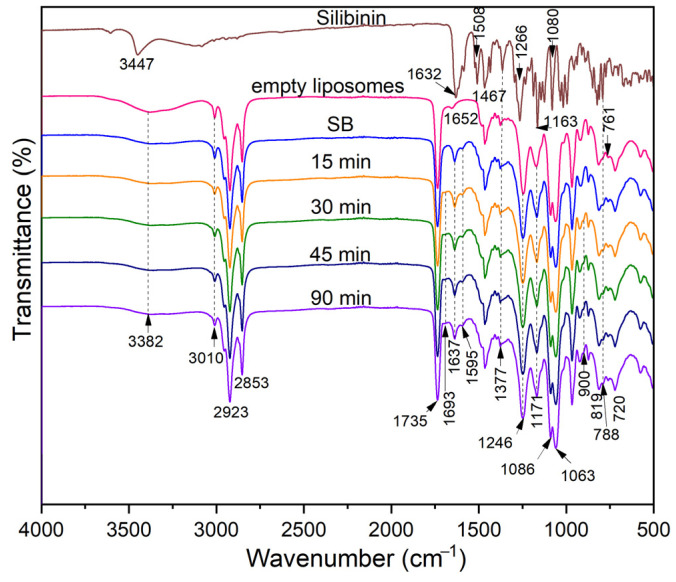
FT-IR spectra of silibinin, empty liposomes, silibinin-loaded liposomes (MLVs), and UV-treated liposomes with silibinin for different periods of 15–90 min of UV irradiation.

No observable differences were found by comparison of the FT-IR spectra of Ph and liposomes, for both empty liposomes and MLVs with silibinin (Figure S1 and Figure 2). It means that the established intra/intermolecular interaction in the packed structure of phosphatidylcholine is not reflected in observable peak shifting or intensity change. Moreover, the absence of new modes on the FTIR spectra of silibinin-loaded liposomes in comparison to empty liposomes (Figure 2) indicates that there is no chemical reaction between the silibinin and phospholipids, therefore indicating their compatibility. Figure S3 shows the FT-IR spectra of silibinin, empty liposomes, silibinin-loaded liposomes as MLVs, and UV-treated liposomes with silibinin for different periods (15–90 min of UV irradiation) in the 1550–1800 cm^−1^ spectral region.

The presence of silibinin in liposomes, i.e., the contribution of aromatic structure absorption before and during UV irradiation, does not noticeably bring intensity peak change in the region 1550–1675 cm^−1^. Also, a hardly observable intensity increase of the valley at 1693 cm^−1^ was observed as a result of oxidation/peroxidation processes causing the formation of oxygen-containing functionalities. Allylic hydrogen is activated to radical group reaction reactive oxygen hydroxyl radical [46]. The formation of hydroperoxides and cyclic forms could undergo C–C scission with the formation of an aldehyde that can then react with oxygen to oxidize to carboxylic groups [47]. To make a more visible and measurable peak of interest, the deconvolution of these peaks was performed in absorbance mode, and the obtained results are given in Figure S4.

A similar phenomenon and trend were observed for both Ph and liposome, indicating that analogous processes but at different intensities take place in these systems. There is a known dependence between two more or less concomitant processes: the disappearance of double bond (Figure S1) and peak structure/intensities change at ~1652 and ~1702 cm^−1^ because of ethylenic bond oxidation/hydroperoxidation by forming oxygen reach species of low stability. The final step is the structural rearrangement to, mainly, aldehyde groups, which easily, under oxidative conditions, transform into carboxylic groups. A similar conclusion can be drawn from the deconvoluted spectra of MLVs with silibinin and their UV-irradiated parallels (Figure S5).

In this way, silibinin can act as a crosslinking agent for phosphatidylcholine by intra-molecular cross-linking being incorporated into a hydrophobic bilayer. At the same time, the phosphatidylcholine protects silibinin from external sources, and thus, only the structural transformation of phosphatidylcholine was noticed.

**Figure 3:**
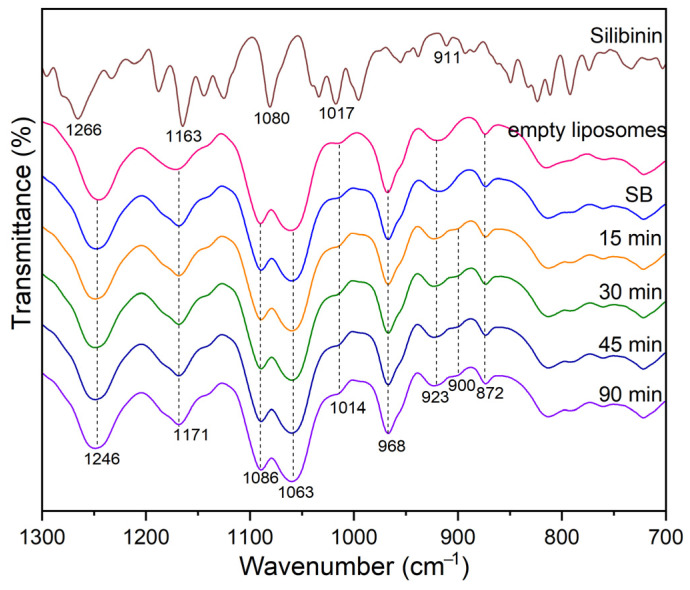
The UV-initiated time-dependent change of the peaks in the 700–1300 cm^−1^ region for silibinin-loaded liposomes.

A similar analysis was applied for the UV-initiated structural change of the studied systems phosphatidylcholine, empty liposomes (Figure S6), and silibinin-loaded liposomes (Figure 3). Here, it was not possible to perform the deconvolution procedure.

The UV irradiation of Ph, liposomes, and silibinin-loaded liposomes leads to spectral change due to the appropriate structural change of treated material (exemplified for silibinin-loaded liposome):

(1) The disappearance of the ethylenic bond was observed as a decrease in the peak intensity at 3010 cm^−1^ (Figure 2),
(2) The change of the peak structure in the region 1600–1750 cm^−1^ related to carbonyl groups stretch vibration of different origins (ester, carboxy, aldehyde carbonyl, etc.) (Figure S3),
(3) The appearance of small shoulder peaks in the region 800–1300 cm^−1^ (Figure S6 and Figure 3).

Due to hardly observable peaks of shifting/intensity change or the appearance of new ones, created because of UV-initiated radical reaction causing the chemical transformation, two methods were applied in this study: the deconvolution of selected peaks and the quantification of peaks area as a measure of the appropriate group presence in the studied molecule.

No noticeable change of the peak at 1735 cm^−1^, originating from the ester carbonyl group of phospholipids, indicates an appropriate stability against oxidative attack. Thus, the height/area of the peak was used as the internal standard value for the calculation of the relative intensities of the peak change. A small noticeable shoulder at 900 and 800 cm^−1^ (Figure S1) indicates the presence of hydroperoxide species, but the position and intensities of other peaks were unchanged. The results obtained based on the applied deconvolution procedure are given in Table S1.

The increased content of oxygen-containing functionalities was reflected as the peak area increased (the peak centered at 1702 cm^−1^) concerning time (30 min). An analogous trend was observed for the silibinin-loaded MLVs system at a lower extent, which indicates the scavenging capability of present silibinin in liposomes.

**Figure 4:**
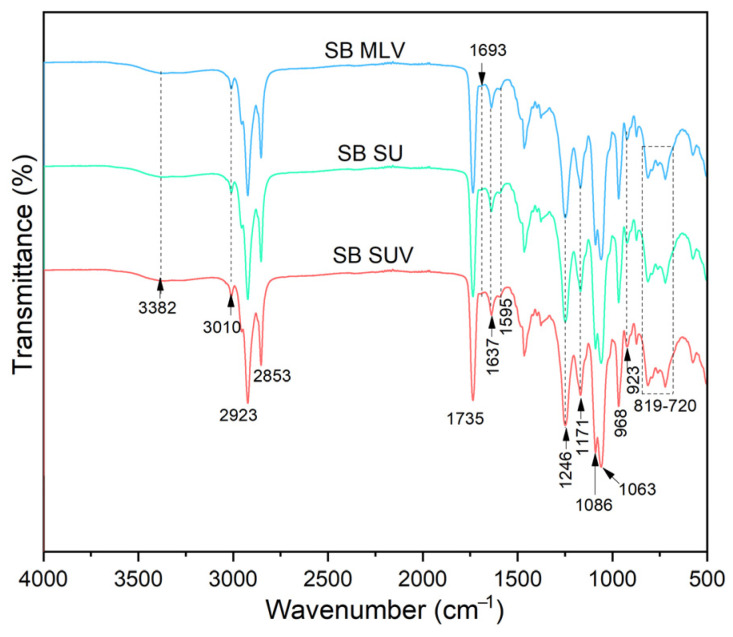
FT-IR spectra of silibinin-loaded liposomes: multilamellar liposomes (MLV), UV-irradiated liposomes, and small unilamellar liposomes (SUV).

As can be seen from Figure 4, sonication did not cause changes in the FTIR spectra of silybin-loaded liposomes. Although ultrasound waves can affect the physical and structural characteristics of liposomes, the degree of the changes depends on ultrasound parameters [48]. In the case of liposomes with silibinin, the sonication period was short (15 min) and included pauses in sonication, which probably prevented the degradation of the bilayer caused by cavitation with a desired reduction in particle size. A prolonged time of ultrasound exposure can show the phase change expanding effect of the phospholipid membrane, and, consequently, chemical and physical changes [48].

Several studies have shown that silibinin is a potent sensitizer of UVA radiation-induced oxidative stress and apoptosis and provides strong protection against UV-induced damage in the epidermis [4,5,13,49,50]. Also, considering that silibinin was encapsulated in liposomes as a carrier that has constituent components potentially sensitive to UV irradiation, sonication, and lyophilization, the stability and chemical changes of free silibinin after UV irradiation, lyophilization, and ultrasound treatment were not examined. The first-mentioned process (UV irradiation) was not used for the treatment of free silibinin because studies have shown not only its stability under UV irradiation but also its protective effects [4,5,13,49,50], and the second-mentioned process (freeze-drying) did not apply to the free silibinin because it is not a common procedure used for a powdered component such as silibinin. Additionally, the sonication of silibinin as a powder would not be feasible using the ultrasonic probe that was employed for liposome sonication.

### 3.6. Raman Spectra

**Figure 5:**
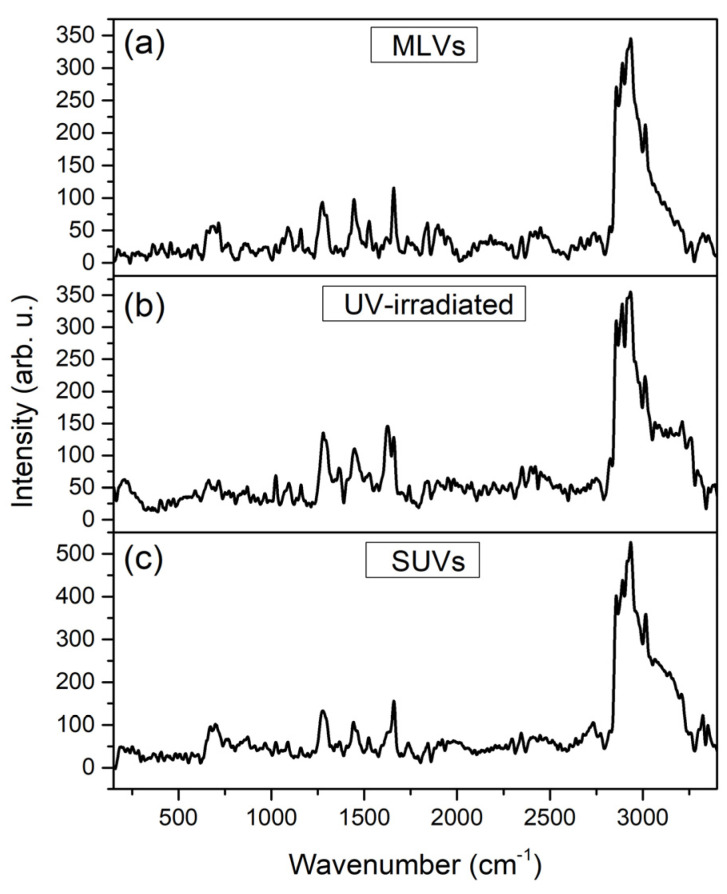
Raman spectra of (**a**) multilamellar vesicles with silibinin (MLVs), (**b**) UV-irradiated liposomes with silibinin, and (**c**) small unilamellar vesicles with silibinin (SUVs).

Raman spectroscopy was applied to investigate the presence of various interactions between silibinin, Ph, liposomes with loaded silibinin, and liposome UV-irradiated and sonicated counterparts. The Raman spectra of pure silibinin and Ph are presented in the Supplementary Materials (Figure S1), while the Raman spectra of MLVs, UV-irradiated liposomes, and SUVs with encapsulated silibinin (all lyophilized samples due to device requirements) are shown in Figure 5.

The Raman spectra of MLVs with encapsulated silibinin (Figure 5a) mostly resembled Ph (Figure S7A), with both showing characteristic bands of phospholipids. When encapsulates almost exclusively show peaks originating from the carrier, i.e., the liposomal bilayer in the case of silibinin-loaded liposomes, it indicates the efficient entrapment of the active compound [51]. The Raman features of the MLVs with silibinin and Ph spectra (Figure 5a and Figure S7A) correspond to the presence of the esters of palmitic and stearic acids, bands between 400 cm^−1^ and 500 cm^−1^ [52], phospholipid head-group C–N symmetric stretching of choline at ~720 cm^−1^ [53], the most characteristic feature of stearic acid at ~850 cm^−1^ [52], C–C=O stretching at ~980 cm^−1^ [45], the skeletal stretching of the C–C vibrations at ~1025 cm^−1^ [53], the symmetric stretching of PO_2_^−^ group at 1080 cm^−1^, and the asymmetric stretching region of the PO_2_^−^ at ~1200 cm^−1^ [54]. The C–H mode from the lipid acyl chains of phospholipids can be related to the *in-plane* CH_2_ twisting mode in the oleoyl chain (1280 cm^−1^) and CH_2_ scissoring mode of the fatty acid chain (1450 cm^−1^) in both MLVs and Ph spectra [55,56]. The band at ~1525 cm^−1^ corresponds to the N-O stretching, while the mode at ~1670 cm^−1^ is related to the C=C stretching vibration in both spectra [53]. The peak at ~1750 cm^−1^ can be associated with the carbonyl group (C=O) of the ester bond among glycerol and fatty acids [57] (Figure 5a and Figure S7A). The peaks in a region of 2100–2750 cm^−1^ in MLVs spectra originate from phospholipids with the fact that they are of a more pronounced intensity than in the case of Ph spectra. The Raman mode in MLVs and Ph spectra at ~2850 cm^−1^ can be related to the symmetric and asymmetric stretching of the C–H bonds of CH_2_ and CH_3_ groups in the alkyl chains, whereas the band at 3015 cm^−1^ can correspond to the CH stretching of the N-CH_3_ [53,57]. The band at ~3350 cm^−1^ is associated with bound water in both spectra [53]. The obtained Raman spectra of pure silibinin (Figure S2B) is in accordance with the literature data in a range of 500–1700 cm^−1^ and 3000–3500 cm^−1^ [58,59], while peaks in a region from 2000 to 2800 cm^−1^ can originate from impurities. As can be seen from Figure 1B, UV irradiation has caused changes in the Raman spectra of silibinin-loaded liposomes. Namely, changes can be noticed in the region of 500–1600 cm^−1^ in the peaks’ intensity (higher intensity in UV-irradiated sample). The phenomenon shown is in agreement with the literature data where there were peaks with strongly higher intensities in the UV-irradiated liposomes in comparison to their non-treated counterparts [33]. In FTIR spectroscopy, the changes are visible in a region of 800–1300 cm^−1^. In addition, the structure of peaks at 1600–1750 cm^−1^ was changed after UV irradiation, which is proven in FTIR analysis as well. The changes are also visible in the region at around 2000 cm^−1^ and 3100–3250 cm^−1^. The mode at 3015 cm^−1^ in MLVs spectra was moved below 3000 cm^−1^ after UV irradiation. The FTIR analysis showed changes in the same region. Ultrasound treatment of silibinin-loaded liposomes did not lead to a change in the Raman spectra, except in terms of peaks’ intensity (Figure 5). Namely, Chotphruethipong et al. [60] reported that the intensity of the peaks of CH_2_ stretching and the C=O group of liposomes (modes at 2800–2900 cm^−1^ and ~1750 cm^−1^, respectively) increased and shifted to a higher wavenumber due to the oxidation of unsaturated fatty acids in the phospholipid membrane. The reason for the absence of the shift of peaks to a higher wavenumber in the case of liposomes with silibinin can be the antioxidant potential of silibinin. Namely, polyphenol compounds are observed to be effective at inhibiting lipid oxidation [48].

### 3.7. ABTS and DPPH Radical Scavenging Potential of Silibinin-Loaded Liposomes

**Figure 6:**
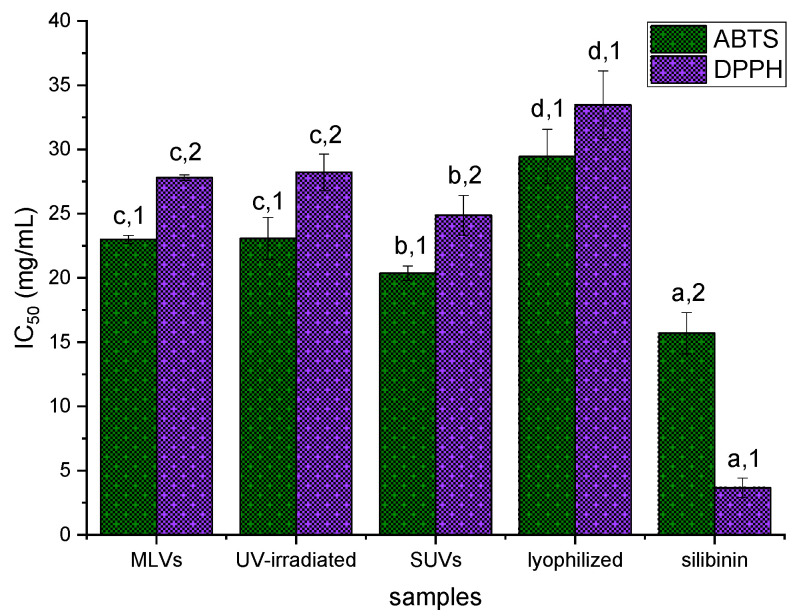
Antioxidant potential of multilamellar vesicles with silibinin (MLVs), UV-irradiated liposomes with silibinin, small unilamellar vesicles with silibinin (SUVs), lyophilized liposomes with silibinin, and pure silibinin; values with the same letter for each assay separately and the same number in each sample separately showed no statistically significant difference (*p* > 0.05; n = 3; analysis of variance, Duncan’s *post hoc* test); IC_50_ (mg of silibinin/mL of liposomal suspension) represented the concentration required to neutralize 50% of free radicals.

Liposomal vesicles can be employed as carriers for antioxidant compounds with the aim of increasing their bioavailability and providing controlled release, while liposome oxidation can be prevented [61]. Therefore, the antioxidant potentials of MLVs, UV-irradiated liposomes, lyophilized liposomes, and SUVs with encapsulated silibinin were investigated via ABTS and DPPH radical scavenging capacity tests, as well as in the cell line with generated ROS (described in Section 3.9). The data from ABTS and DPPH assays are presented in Figure 6.

The ABTS radical scavenging activity of developed liposomes with silibinin, expressed as the IC_50_ value, was 22.99 ± 0.32, 23.08 ± 1.62, 20.36 ± 0.56, and 29.44 ± 2.13 mg of silibinin/mL of liposomal suspension for MLVs, UV-irradiated liposomes, SUVs, and lyophilized liposomes, respectively (Figure 6). The antioxidant capacity determined in the DPPH assay, expressed as the IC_50_ value, was 27.80 ± 0.21, 28.21 ± 1.42, 24.86 ± 1.54, and 33.46 ± 2.64 mg of silibinin/mL of liposomal suspension for MLVs, UV-irradiated liposomes, SUVs, and lyophilized liposomes, respectively (Figure 6). The determined IC_50_ value of ascorbic acid, as a control, was 0.217 mg/mL in the ABTS test, and 0.052 mg/mL in the DPPH test. UV irradiation did not cause changes in the antioxidant potential of liposomes measured in both employed assays, while the lyophilization process significantly decreased the radical scavenging activity of liposomes with silibinin. In contrast, sonication positively influenced the antioxidant capacity of liposomes with silibinin (lower IC_50_ value = higher antioxidant potential) (Figure 6). The antioxidant potential of pure silibinin was 15.69 ± 1.61 and 3.65 ± 0.76 mg of silibinin/mL of ethanol, in ABTS and DPPH assays, respectively (Figure 6).

### 3.8. Cytotoxicity of Silibinin-Loaded Liposomes

**Figure 7:**
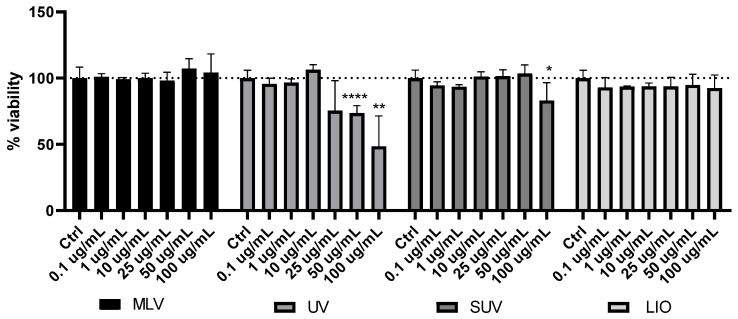
Cytotoxicity of multilamellar liposomes (MLVs), UV-irradiated liposomes, lyophilized liposomes, and small unilamellar liposomes (SUVs) with encapsulated silibinin in a range of concentrations (0.1, 1, 10, 25, 50, and 100 µg/mL) determined by MTT assay in HaCaT cells. Data are expressed as mean + SEM relative to the unexposed control (dashed line); * *p* < 0.05, ** *p* < 0.01, **** *p* < 0.0001 by one-way analysis of variance (ANOVA) with Tukey’s multiple comparison *post hoc* test.

In view of silibinin activities toward skin presented in various studies [4,5,13], the effect of developed liposomes on keratinocyte viability was examined. Figure 7 graphically shows the results obtained.

Figure 7 represents the effect of MLVs, UV-irradiated liposomes, lyophilized liposomes, and SUVs with encapsulated silibinin on cell viability in HaCaT cells. The treatment of cells with the liposomes for 24 h produced diverse effects on cells, depending on the type of liposomes. Namely, MLVs and lyophilized samples did not lead to a significant change in cell viability compared to the unexposed control in any of the concentrations used. On the other hand, the UV-irradiated sample showed cytotoxic effects in concentrations above 25 µg/mL and significantly reduced cell viability at 50 and 100 µg/mL in a concentration-dependent manner, where a greater reduction in cell viability was observed with increasing concentrations. Finally, SUVs showed a significant decrease in the percentage of live cells in the treatment with the highest concentration of 100 µg/mL, compared to the cells exposed to medium alone (control). The cytotoxic influence of non-loaded liposomes (data in Supplementary Materials, Figure S8) was also noticed but using a concentration of phospholipids of 1000 µg/mL.

### 3.9. Antioxidative Effect of Silibinin-Loaded Liposomes in H_2_O_2_-Induced Oxidative Stress

**Figure 8:**
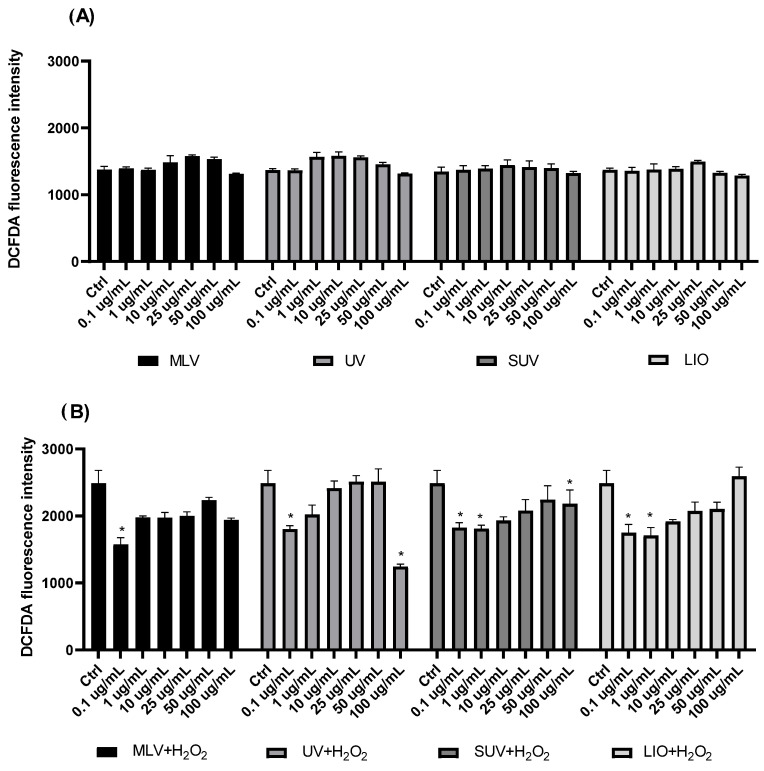
Effect of 24 h pre-incubation with multilamellar liposomes (MLVs), UV-irradiated liposomes, lyophilized liposomes, and small unilamellar liposomes (SUVs) with encapsulated silibinin in a range of concentrations (0.1, 1, 10, 25, 50, and 100 µg/mL) on the production of reactive oxygen species in HaCaT cells *versus* control (**A**) without H_2_O_2_ and (**B**) after the exposure to H_2_O_2_; determined by H2DCFDA assay, expressed as relative fluorescence intensity. The data are expressed as mean + SEM; * *p* < 0.05 by one-way analysis of variance (ANOVA) with Tukey’s multiple comparison *post hoc* test.

The antioxidative effect of MLVs, UV-irradiated liposomes, lyophilized liposomes, and SUVs with encapsulated silibinin on H_2_O_2_-induced oxidative stress in HaCaT cells was investigated as well. The obtained data are presented graphically in Figure 8.

Figure 8 represents the effects of 24 h treatment with MLVs, UV-irradiated liposomes, lyophilized liposomes, and SUVs with encapsulated silibinin on the levels of ROS in the HaCaT cells. The analysis of the effects of all liposomal samples (Figure 8A), after 24 h incubation in the HaCaT cells without exposure to H_2_O_2_, showed that all examined types of liposomes with silibinin did not significantly alter endogenous ROS production. It should also be mentioned that empty liposomes were tested for ROS production in the HaCaT cells after 24 h, and the results showed that the highest concentration of phospholipids (1000 µg/mL, empty liposomes, i.e., phospholipid liposomes without silibinin) induced an elevated production of ROS, while smaller concentrations did not affect ROS levels (data in Supplementary Materials, Figure S9).

After exposure to 200 µM H_2_O_2_ for 2 h, the production of ROS was elevated almost two-fold in HaCaT cells (H_2_O_2_ bar in Figure 8B). The treatment by pre-incubation of cells with the silibinin-loaded liposomes resulted in reduced ROS levels compared to cells exposed to H_2_O_2_ alone in a concentration-dependent manner, where smaller concentrations were more efficient in reducing ROS. MLVs and UV-irradiated liposomes with silibinin showed a significant decrease in the ROS levels at 0.1 µg/mL concentration, while SUVs and lyophilized liposomes with silibinin showed a reduction at 0.1 and 1 µg/mL. Additionally, UV-irradiated liposomes and SUVs with silibinin also displayed a significant decrease in ROS levels at the highest concentration of 100 µg/mL.

### 3.10. Anti-Inflammatory Potential of Silibinin-Loaded Liposomes

Since it is known that COX-2, IL-1β, and MIF play important roles in the regulation of inflammatory response in skin cells, the effects 24 h pre-incubation with MLVs, UV-irradiated liposomes, lyophilized liposomes, and SUVs with encapsulated silibinin at a final concentration of 10 µg/mL were examined in cells challenged with LPS.

**Figure 9:**
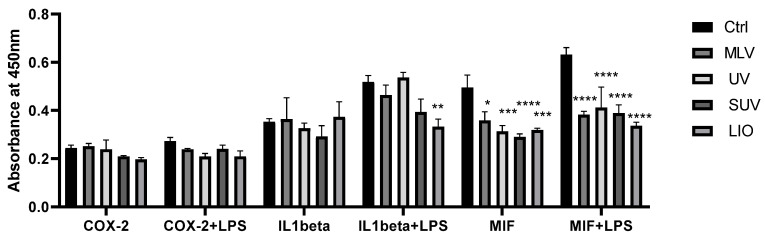
Effect of 24 h pre-treatments with multilamellar liposomes (MLVs), UV-irradiated liposomes, lyophilized liposomes, and small unilamellar liposomes (SUVs) with encapsulated silibinin at final concentration of 10 µg/mL in complete medium on the protein expression of cyclooxygenase-2 (COX-2), interleukin 1 beta (IL-1β), and macrophage inhibitory factor (MIF) in HaCaT cells, with or without lipopolysaccharide (LPS) exposure, using the cELISA method; * *p* < 0.05, ** *p* < 0.01,*** *p* < 0.001, **** *p* < 0.0001 by one-way analysis of variance (ANOVA) with Tukey’s multiple comparison *post hoc* test.

The results presented in Figure 9 show that LPS treatment induced a significant elevation of IL-1β and MIF, and only a slight rise in COX-2 levels in LPS-exposed cells compared to non-treated cells. In cells exposed to liposomes alone, without LPS, there was a moderate inhibition of COX-2 expression in HaCaT cells treated with SUVs and lyophilized liposomes and inhibition of IL-1β by SUVs. The results showed that plain liposomes (phospholipid liposomes without silibinin) at a concentration of 10 µg/mL did not induce protein expression of IL-1β, MIF, and COX-2 in comparison to the control, i.e., treatment without liposomes (data in Supplementary Materials, Figure S10). Furthermore, all four types of liposomes significantly reduced MIF expression in cells incubated with liposomes alone for 24 h. Next, in cells exposed to LPS, there was a significant difference in MIF levels at pre-treatments with liposomes at 10 µg/mL compared to LPS alone, confirming the inhibitory effect of MLVs, UV-irradiated liposomes, lyophilized liposomes, and SUVs with encapsulated silibinin on MIF expression after the LPS challenge. Considering IL-1β levels in cells treated with LPS, pre-incubation of lyophilized liposomes significantly reduced the protein expression, while other types of liposomes did not show significant change, although SUVs also showed a reducing trend. Since COX-2 levels were not significantly elevated after LPS treatment, the change in pre-treatments with liposomes could not be observed.

## 4. Discussion

The EE of different silibinin-loaded liposomes, as a significant parameter of the encapsulation process, follows the following trend: MLVs and UV-irradiated liposomes > SUVs > lyophilized liposomes. A significantly lower EE of the lyophilized liposomal vesicles was expected since the process of freeze-drying is harmful to the integrity of the bilayer membrane. According to the literature data, a decrease in encapsulation efficiency is noticed for lyophilized formulations of liposomes, particularly in the absence of the cryoprotectant, as in the case of silibinin-loaded liposomes developed in the present study. Namely, lyophilization results in liposome degradation caused by ice crystals, destruction of the phospholipid membrane function, and consequently, leakage of encapsulated compounds [60,62]. Prolonged exposure to ultrasound waves can also cause a higher decrease in EE (as in liposomes with silibinin) due to higher cavitation effects and the rupture of the vesicles, consequently leading to a potentially excessive release of encapsulated silibinin. Nevertheless, sonication can be used to improve the EE and stability of the liposomal particles in the presence of protein hydrolysates adhering to the membrane internally [63]. The exposure to UV irradiation did not result in a change in the EE of silibinin since UV rays do not create ruptures on the liposomal bilayer, which agrees with the literature data where UV irradiation did not cause a leakage of encapsulated plant bioactives [33].

The average size of liposomal vesicles is significantly affected by the type of used lipids, the liposomal preparation technique, and the physicochemical characteristics of the encapsulated compounds [16,19,28]. In addition, the appropriate diameter of liposomes is essential in terms of the delivery of active components through the skin since larger vesicles cannot diffuse through the skin layers (stratum corneum and deep skin) and achieve the target location. For example, liposomes with a size of 50 nm show a higher diffusion rate in comparison to the vesicles of 200 nm. Small liposomes (diameter of 120 nm) provided a higher accumulation of encapsulated compounds in the stratum corneum and deeper skin in comparison to bigger particles [29]. In the case of silibinin-loaded liposomes, the mentioned parameter follows the following trend: MLVs and UV-irradiated liposomes > lyophilized liposomes > SUVs. UV irradiation did not affect the diameter of liposomes, whereas sonication caused a significant decrease in the particle size. The size of the lyophilized liposomes shows that the lyophilization process led to a decrease in the size of the liposome particles with silibinin in comparison to MLVs. In the case of smaller liposomes (50–300 nm), the freeze-drying process increases the chance of membrane apposition and the creation of larger particles (fusion/aggregation of vesicles) due to the higher liposome concentration by the propagating ice front and the absence of the hydration barrier to fusion [64]. On the other hand, for larger liposomes (such as the silibinin-loaded liposomes developed in the present research), the fragmentation of particles (decreasing in size) is possible during lyophilization [65]. The obtained value of vesicle size of SUVs agrees with the literature data where the mentioned parameter varied between 250 and 280 nm after the ultrasound treatment of the liposomes due to the efficient conversion of larger multilayered vesicles (multilamellar) into single-layered (unilamellar) ones [63]. Namely, the size of liposomes depends on the number of their layers, which can vary in a very wide range (from nm to mm) in thickness [66]. A short ultrasound treatment period (15 min was used for silibinin-loaded liposomes) was required to obtain nano-sized particles, while prolonged sonication time can cause the complete rupture of the liposomal membrane, causing the leakage of encapsulated components, as well as the easy binding of broken integrity membranes, leading to an increase in particle size [48,63]. Silva et al. [67] have also obtained nano-sized liposomes (~200 nm) after 15 min of sonication. UV irradiation can change the physical properties of liposomal bilayers by disturbing the order and phospholipid packing, as well as causing an increase in membrane fluidity and permeability [68,69]. UV irradiation also excites vibrational motions in molecules of the complex, changing the lengths and angles of bonds and causing electronic transitions and the cleavage of chemical bonds. However, the mentioned changes were not visible when measured immediately after UV radiation of silibinin-loaded liposomes. The encapsulation of silibinin can affect membrane integrity and avoid further disintegration under UV irradiation, due to silibinin incorporation between the two imperfect adjoining chains within lipid bilayers of mono- and polyunsaturated phospholipid chains [69]. Silibinin, as a highly hydrophobic compound, can be “sandwiched” between the two monolayers, providing the stabilization of the membrane structure and preventing extra damage.

The PDI values for MLVs, UV-irradiated, and lyophilized liposomes are ~0.3, indicating the mono-dispersity or homogeneity of the system [42]. PDI also remained around 0.3 for liquid and lyophilized phospholipid liposomes with rutin, showing that freeze drying did not cause the changes in PDI values [70], as in the case of liposomes with encapsulated silibinin. The highest PDI was recorded for the SUVs, which can mean the presence of MLVs along with SUVs. The main disadvantages of sonication, as the most extensively employed technique for the preparation of SUVs, are the low encapsulation efficacy, possible degradation of phospholipids and encapsulated compounds, metal pollution from the probe tip, and presence of MLVs along with SUVs [26]. The measured value of PDI of SUVs agreed with the results of the Silva et al. study [67] where the PDI was ~0.5 after 18 min of sonication. According to the literature data, a higher uniformity of liposomal vesicles can be achieved by prolonged ultrasound treatment or the usage of higher amplitude [63,67]. However, ultrasound waves can promote phospholipid hydrolysis and oxidation via the production of free radicals in the cavitation bubbles collapse, while a high temperature that arises due to long-term sonication can accelerate phosphocholine hydrolysis as well [67]. The Arias-Alpizar et al. study [71] has also shown that the measured size and PDI of liposomes were unchanged before and after UV irradiation. In addition, potential membrane reorganization does not always have to lead to a change in liposome integrity, size, size distribution, or the leakage of liposome-encapsulated compounds during and after UV irradiation, which was also proven by measurement of EE after irradiation (Table 1).

The zeta potential of all tested silibinin-loaded liposomes possessed negative values and reached the highest level (absolute value) in the case of MLVs and UV-irradiated liposomes. The values of the zeta potential of MLVs and UV-irradiated samples were not significantly different. Zeta potential values were negative due to the anionic phospholipids, including phosphatidylcholine, and higher (absolute value) than −30 mV, indicating that the MLVs and UV-irradiated samples are considered stable due to the relatively high repulsive forces, preventing the aggregation, flocculation, or sedimentation of their vesicles [70]. However, freeze drying and ultrasound waves significantly decreased the zeta potential (absolute value) of the obtained liposomes. The decrease in zeta potential after ultrasound treatment can be explained by the release of a small extent of the hydrophobic core or molecules due to the cavitation covering the negatively charged surface [63]. In the freeze-drying process, the temperature changes can decrease the zeta potential value and consequently, the crystal structure of the lipids was altered, and the release of encapsulated compounds from the liposomes was increased (that is also shown in Section 3.1.); in addition, the liposomal stability was reduced [48]. Chotphruethipong et al. [60] have also reported that active compounds plausibly liberated from liposomes can interact with a negative charge of phospholipids that, subsequently, can be partially neutralized, resulting in decreased negative surface charge. Several studies showed that the presence of hydrophobic compounds in the bilayer, particularly on the surface, can mask the negative charge [63,72,73]. The drop in the values of zeta potential after ultrasound treatment and lyophilization can result in decreased repulsive interactions between liposomal particles, low stability, and flocculation occurrence, since the negative charge was below −30 mV [63]. The conductivity of liposomal suspension follows the following trend: lyophilized liposomes > SUVs > UV-irradiated liposomes > MLVs. Since UV irradiation caused water evaporation from the liposome sample, the increase in the conductivity factor in the mentioned formulation should be explained by higher lipid and ion concentrations. On the other hand, a significantly higher conductivity factor of lyophilized liposomes and SUVs should be correlated with a lower EE. Namely, the increase in the conductivity factor in the liposome sample can be related to the release of entrapped components [74]. Since the mobility of liposomes is a function of the diameter, surface charge, and lipid composition of liposome vesicles, as well as the characteristics of encapsulated compounds [75], variations between different liposomal formulations were expected. The lower mobility of SUVs and lyophilized liposomes with silibinin compared to the MLV and UV-irradiated samples can be due to the potentially adsorbed flavonoid compound, such as silibinin, at the surface of the liposomal bilayer. Since ultrasound treatment and lyophilization cause the leakage of encapsulated compounds, which are proven by the lower EE in SUVs and lyophilized samples with silibinin (Table 1), the presence of silibinin from the outer membrane of the liposome is possible. Namely, according to Yang et al. [76], the presence of flavonoids on the liposome surface can result in decreased mobility.

A significant increase in vesicle size was noticed in all developed liposomes with silibinin during 60 days of storage at 4 °C. According to Hamadou et al. [43], the aggregation generated by the accumulation of liposomal vesicles can significantly influence liposome size and distribution. The most prominent increase can be noticed in the lyophilized sample, which showed the lowest absolute value of zeta potential (Table 1), i.e., the lowest potential to prevent the aggregation of particles. In the case of SUVs with encapsulated silibinin, the absolute value of zeta potential lower than 30 mV can be also responsible for a significant increase in particle diameter due to the decrease in repulsive interactions, both electrostatic and steric, creating a system that is prone to accumulation [42]. Additionally, the particle size of UV-irradiated liposomes showed consequential variations and, after the 21st day, their size was significantly higher in comparison to the non-treated parallel, suggesting the photochemical destruction of products due to absorption of photon energy and change in the liposome bilayer conformation [68,69]. Also, the predominant effect of UV light on the bilayer damage, i.e., photodegradation from highly disordered polyunsaturated fatty acids chains of phospholipids and the exposition of hydrophobic patches, could promote particle aggregation during storage time [69]. Moreover, physical factors can influence the shelf life of liposomes, such as aggregation/flocculation and fusion/coalescence, size changes, and drug loss, which represents an important disadvantage of liposome usage. Although lyophilization is suitable for liposomal bilayers with heat-sensitive compounds, the stability of lyophilized liposomes can be lost and depends on the freezing rate, liposome formulation technique, and membrane composition, as well as the residual moisture content. Thus, the optimization of the mentioned factors and/or the utilization of appropriate cryoprotectants can improve the stability, encapsulation efficiency, and biological potential of freeze-dried liposomal particles [77]. The potential strategies to mitigate these negative effects that occur during the freeze drying of liposomes for future studies can include the addition of cryoprotectants, such as carbohydrates (monosaccharides, disaccharides, polysaccharides, or synthetic saccharides), proteins (amino acids), and alcohols, as well as additional encapsulation of active compounds in various carriers, e.g., cyclodextrins (double loading technology). The addition of the mentioned cryoprotectants or cyclodextrins to the liposome formulation can prevent particle aggregation and the leakage of the encapsulated compounds and protect the liposomal bilayer from degradation caused by ice crystals [77,78]. The PDI value of the lyophilized sample showed a slight increase in heterogeneity, while the PDI significantly increased in MLVs and UV-irradiated and SUV liposomal suspensions, indicating less homogeneity and more aggregation. The PDI significantly affects the physical stability of the liposomal suspension and, therefore, the value should be as low as possible to provide the long-term stability of the nanosuspension vesicle size distribution [42]. A significant decrease in the zeta potential (absolute value) was noticed in all liposomal forms during the 60-day storage study. The Lopez-Polo et al. study [70] reported that the zeta potential of liquid and lyophilized liposomes (absolute value) decreased during storage at 4 °C, particularly in the case of dried form. All developed liposome formulations have retained negative values of zeta potential in the 60-day storage study. Since the polar heads or phosphate groups of phospholipids (mainly phosphatidylcholine) are responsible for the negative charge of the surface, it can be concluded that the reorganization of phospholipids in the lipid bilayer did not occur over time [63,72]. In addition, the decrease in zeta potential values during time can be explained by the size changes. Namely, according to the literature, the charge can be associated with the liposomal vesicle size, and the smallest size is correlated with a larger surface area, providing the exposure of the phosphate groups to the aqueous surrounding, which results in an increased negative charge [63]. Considering that over time, the particle size of all developed liposomes was significantly increased (Figure 1A), the values of zeta potential consequently decreased due to the lower surface area. The surface charge of liposomes might be partially neutralized via the interaction of negatively charged phospholipids with potentially released compounds, which lead to the enhanced aggregation of liposomal particles [60]. It was also evidenced by the decreased zeta potential and increased diameter of the vesicles of silibinin-loaded liposomes. In the 60-day storage study, the conductivity significantly increased in MLVs and SUVs with silibinin, whereas the mentioned parameter was not changed in the UV-irradiated sample. A significant drop in the conductivity of the lyophilized liposomes might be explained by the greater surface area (smaller liposomal vesicles on the 1st day) exposing a greater percentage of head groups of phospholipids that most notably affected the conductivity (higher conductivity factor on the 1st day) in comparison to larger vesicles at the end of the storage study [79]. The mobility of MLVs and UV-irradiated lipid vesicles with silibinin significantly decreased during storage, while the decrease in the mobility of SUVs and lyophilized samples was more gradual. A significant increase in vesicle size during storage (Figure 1A) can explain the drop in the mobility of all prepared liposomal formulations. Yanagihara et al. [80] have reported that liposomes’ size affects their migration behavior in tissues, cells, and blood circulation.

A significantly lower density of liposomal suspension after UV irradiation and ultrasound treatment is probably due to the occurrence of hydrolytic reactions in aqueous surroundings. In addition, decreased density results in higher fluidity and less stability, which is important for the application in preparations that are intended for longer usage and storage [81,82]. The obtained values of surface tension are higher in comparison to the surface tension of different liposomes in the literature [53,83], probably due to the presence of various lipids in their liposomal bilayer, such as lecithin, which can have the role of surfactant and variations in the characteristics of encapsulated compounds. Since flavonoid compounds can be good stabilizers of emulsion systems, due to their adsorption at the surface [84], and SUVs showed a higher amount of silibinin in aqueous surroundings (lower EE, Table 1), it can explain the significantly lower surface tension in the mentioned sample. All developed liposomes with silibinin showed very low viscosity. According to the literature data, liposomal suspension with lower viscosity showed a significant change in the vesicle size of liposomes [81], which was also proven in the stability study of silibinin-loaded liposomes (Figure 1A). Based on Stoke’s law, viscosity values are in reverse relation with the sedimentation of vesicles, i.e., an increment in viscosity can decrease the rate of sedimentation; the size distribution of high viscosity liposomes remained unchanged for a longer time and, therefore, more viscous formulations are more stable [81]. Thus, in the case of silibinin-loaded liposomes, viscosity modifiers should be used to decrease the chance of size separation and changes, as well as sedimentation.

UV irradiation did not cause significant changes in the ABTS and DPPH radical scavenging potential of silibinin-loaded liposomes, confirming the protective role of liposomal particles on bioactive compounds. On the other hand, freeze drying significantly decreased the antioxidant capacity of silibinin-loaded liposomes. A slow freezing rate can damage the lipid bilayer due to the formation of large ice crystals and induce deformations via mechanical stresses and osmotic pressure, resulting in the release of encapsulated antioxidants, i.e., lower EE [77] which is also determined in the case of silibinin-loaded liposomes (Table 1). Among all developed liposomes in the present study, the lyophilized sample possessed the lowest amount of encapsulated silibinin. In addition, since the antioxidant tests were performed after the reconstruction of lyophilized liposomes in water, it can lead to the rearrangement of the liposome structure, as well as the incomplete re-suspension of phospholipid particles, which can cause a decrease in the overall antioxidant potential of the liposomes [77]. Although SUVs also showed lower EE compared to MLVs and UV-irradiated liposomes, the effect of ultrasound waves positively influenced the antioxidant potential of liposomes with silibinin, probably due to a larger contact surface of smaller liposomal particles with free radicals. The antioxidant activity of pure silibinin was higher in both antioxidant tests. Therefore, it can be noticed that the liposome surroundings significantly influenced the antioxidant capacity of silibinin.

MLVs and lyophilized liposomes with silibinin did not cause a significant change in the viability of the HaCaT cells, while the UV-irradiated parallel possessed cytotoxic effects. On the other hand, SUVs showed a significant decrease in the viability of HaCaT cells. The obtained result can be related to potential free radicals and lipid peroxidation in liposomal suspension that can be produced due to UV irradiation and ultrasound treatment. UV irradiation caused the chemical changes in developed liposomes that were proven in the FT-IR and Raman analyses (Section 3.5 and Section 3.6). Namely, free radicals (produced by ultrasound probe or under UV irradiation) can change the protein structure and induce apoptosis and the release of cytokines responsible for inflammatory reactions in the skin [85]. Free radicals trigger different biological responses via the activation of transcription factors as well, while lipid peroxidation induces the expression of vascular endothelial growth factors in human keratinocytes [85,86]. In the case of free radical-induced lipid oxidation, ferroptosis is a recognized form of programmed cell death different from apoptosis, necroptosis, and pyroptosis. According to the literature, ferroptosis is the basis of the pathogenesis of various skin diseases, including psoriasis, collagen diseases, and skin cancers [87]. In the previous paper, it was shown that UV-irradiated liposomes possessed Raman spectra bands with strong intensities located at 834 and 867 cm^−1^ as the most characteristic features of stearic acid [33]. In addition, the current study reported that FT-IR analysis confirmed the presence of bands in the spectra between 800 and 900 cm^−1^, indicating the presence of hydroperoxide species for the UV-irradiated liposomes, a potentially oxidative derivative of stearic acid. In various papers, it was shown that stearic acid and its oxidative derivative are lipotoxic, decrease cell viability, and induce cell death [88,89]. This agrees with the observation related to the UV-irradiated liposomes in HaCaT cells, and it is plausible that an increase in stearic acid and its oxidative derivatives content confer their elevated cytotoxicity. The observed cytotoxic effect of empty liposomes can be explained by the excessive amount of phospholipids, which probably caused this cytotoxicity. It corresponds to what other authors observed, e.g., that cell death in HaCaT keratinocytes could be induced by the presence of large amounts of lecithin in the formulations [90]. In the study with the HEK-293 cells, the reduction in the cells’ viability was perceived at the highest-used concentrations of lecithin-based systems: emulsions, liposomes, and aqueous lecithin dispersion (at 10% or 25%), while formulations diluted 10-fold (to a final phospholipid concentration of 0.12% and 0.5% in emulsions and other dispersions, respectively), did not cause any toxic effect on the cells [91]. According to the literature data, one of the main disadvantages of liposome usage is their degradation when the hydrocarbonate chains hydrolysate, the ester bond, to glycerol, and by the peroxidation of unsaturated chains, leading to short-chain lipids, which will form soluble derivatives, decreasing the quality of the liposomal system [29].

The cell treatment using liposomes with silibinin reduced ROS levels in comparison to cells exposed to H_2_O_2_ alone. The Li et al. study [92] showed that silibinin can decrease the mitochondrial ROS level. On the other hand, silibinin has been found to increase the ROS production involved in apoptosis and induce oxidative stress in cancer cell lines [93]. Since MLVs and all modified liposomal systems with silibinin showed a significant decrease in the ROS levels at the same concentration (0.1 µg/mL), it can be concluded that the modification technique did not have a significant influence on the antioxidant potential of the obtained liposomes. However, UV-irradiated liposomes and SUVs caused a significant decrease in ROS levels at the highest concentration. This was probably due to the smaller cell number producing ROS, considering that these concentrations of UV-irradiated liposomes and SUVs with silibinin reduced the cell viability as shown in MTT (Figure 7). Namely, when unsaturated and other lipids are present in liposomal vesicles, photon energy emissions during UV irradiation can result in membrane disorders, due to the formation of free radicals through various processes, including one-electron redox reactions, thermal homolysis of the bonds, and high-energy radiation, as well as photolysis [69].

All developed liposomes with silibinin significantly reduced MIF expression in cells incubated for 24 h only with liposomes (without LPS). The results from the experiment where the cells were exposed to LPS confirmed the inhibitory effect on the MIF expression of all four liposomal forms. Namely, all tested liposomes with silibinin (non-modified and modified samples) significantly influenced the MIF expression, causing its inhibition. Thus, post-processing procedures for the liposomal modifications did not significantly affect the inhibitory capacity of the developed silibinin-loaded liposomes in terms of the MIF level. The Ramasamy et al. study [94] showed that silibinin decreased the level of MIF in tumor-associated macrophages. The pre-incubation of lyophilized liposomes significantly reduced the expression of IL-1β (in cells treated with LPS), whereas SUVs showed a reducing but not significant trend. In contrast, MLVs and UV-irradiated liposomes did not have a significant influence. Namely, the liposome size and its reduction (SUVs and lyophilized samples possessed a lower diameter, Table 1) play a significant role in terms of skin delivery because the mentioned parameter influences the penetration of encapsulated components through the skin to the deeper layers [29]. For example, the reduced particle size of liposomes was used to enhance their therapeutic efficacy in vitiligo [29]. The Peralta et al. study [95] has shown that liposomes with an average vesicle size of 100 nm function effectively as enhancers of skin penetration, consequently improving the efficiency of the applied preparation. Since silibinin has reduced the level of IL-1β in preeclamptic women, exhibiting potent anti-inflammatory activity [96], the reason for the absence of a significant down-modulation of inflammatory cytokine production, such as IL-1β, can be explained by its encapsulation in liposomal particles and potential prolonged or postponed release. In the case of lyophilized liposomes and SUVs with silibinin, the particle size was significantly lower in comparison to MLVs and UV-irradiated parallels; thus, a higher release of encapsulated silibinin due to a higher contact surface can be the reason for the better effect on the reduction of the IL-1β expression. The Yan et al. study [97] demonstrated that silibinin in liposomes had better effects on inflammation than silibinin alone in an in vivo model via modulating signaling pathways, but after oral and parenteral applications, due to excellent oral absorption and bioavailability.

## 5. Conclusions

Silibinin, as a potent antioxidant, antimicrobial, anti-inflammatory, and UV protective agent, was encapsulated in phospholipid liposomes to improve its stability and bioavailability. The liposomes obtained were further modified via UV irradiation, sonication, and lyophilization. The effect of UV irradiation, the ultrasound reduction of vesicle size, or freeze drying on the cytotoxic, antioxidant, and anti-inflammatory capacity of liposomes with encapsulated silibinin on keratinocytes, storage stability, and the FTIR and Raman fingerprint spectra were investigated for the first time in the present research. Different treatments of silibinin-loaded liposomes caused diverse effects on physicochemical properties and biological activities, depending on the type of the process. UV irradiation significantly changed the rheological characteristics of the liposomes and increased the cytotoxic effect on HaCaT cells due to chemical changes proven by FTIR and Raman spectroscopy. The freeze-drying process significantly affected the physical characteristics of liposomes, decreased their EE and ABTS and DPPH radical scavenging activity, and enhanced their anti-inflammatory potential (reduction of the expression of potent proinflammatory cytokine, IL-1β, in HaCaT cells treated with LPS). The sonication significantly decreased the EE and changed the physical and rheological characteristics of silibinin-liposomes, and slightly increased their cytotoxicity. On the other hand, the inhibitory effect on the expression of IL-1β and anti-ABTS and the anti-DPPH capacity of sonicated liposomes was significantly enhanced. All silibinin-loaded liposomes showed an increasing trend in particle size and a decreasing trend in zeta potential (absolute values) during storage. MLVs and lyophilized liposomes with silibinin showed promising antioxidant potential on ROS generated in HaCaT cells and anti-inflammatory activity via reducing MIF expression in HaCaT cells treated with LPS, and did not cause a cytotoxic effect. Due to the shown promising bioactivities related to skin cells and the possible synergistic beneficial effects of silibinin and phospholipids on human skin, the developed liposomal systems can find application in various cosmetic or pharmaceutical formulations. However, UV irradiation should not be used as a method for the sterilization of silibinin-loaded liposomes because of the shown cytotoxic potential of the obtained samples. Nevertheless, freeze drying can be employed as a technique for the prevention of hydrolytic and oxidative degradation in a final silibinin-liposome formulation due to preserved bioactivities (antioxidant and anti-inflammatory) and the absence of cytotoxic effect. Therefore, future perspectives will be focused on the optimization of the lyophilization process via varying the pressure, temperature, and time of the process, employing various types of cryoprotectants and their amounts, as well as on wound healing, anti-aging, and other potential effects of silibinin-loaded liposomes in sophisticated cell-based models of skin diseases, wounds, and aging.

## Figures and Tables

**Table 1:** The encapsulation efficiency (EE), particle size, polydispersity index (PDI), zeta potential (ζ), conductivity (presented as the conductivity factor, CF), and mobility (μ) of multilamellar liposomes (MLVs), UV-irradiated liposomes, lyophilized liposomes, and small unilamellar liposomes (SUVs) with encapsulated silibinin, and unloaded MLVs.

Samples	EE [%]	Particle Size [nm]	PDI	ζ [mV]	CF	*μ* [µmcm/Vs]
MLVs with silibinin	89.7 ± 1.4 ^a,^*	1675.0 ± 44.3 ^a^	0.310 ± 0.019 ^b^	−35.5 ± 0.7 ^a^	0.38 ± 0.01 ^d^	−2.78 ± 0.02 ^a^
UV-irradiated with silibinin	88.1 ± 1.2 ^a^	1701.5 ± 58.7 ^a^	0.272 ± 0.034 ^b^	−36.5 ± 0.7 ^a^	1.15 ± 0.02 ^b^	−2.89 ± 0.07 ^a^
Lyophilized with silibinin	62.5 ± 1.9 ^c^	724.9 ± 27.5 ^c^	0.334 ± 0.031 ^b^	−14.9 ± 0.5 ^c^	2.64 ± 0.30 ^a^	−0.70 ± 0.06 ^c^
SUVs with silibinin	74.9 ± 1.0 ^b^	277.8 ± 2.7 ^d^	0.520 ± 0.059 ^a^	−21.6 ± 0.1 ^b^	1.24 ± 0.08 ^c^	−1.58 ± 0.04 ^b^
Unloaded MLVs	n.a.	1435.8 ± 22.1 ^b^	0.287 ± 0.022 ^b^	−10.3 ± 0.4 ^d^	0.32 ± 0.02 ^e^	−0.51 ± 0.03 ^d^

* Values with the same letter in each column showed no statistically significant difference between different developed liposomes with silibinin (*p* > 0.05; n = 3; analysis of variance, Duncan’s *post hoc* test); 1 CF = 10 μS/cm; n.a., not applicable.

**Table 2:** The density (ρ), surface tension (γ), and viscosity (η) of multilamellar liposomes (MLVs), UV-irradiated liposomes, and small unilamellar liposomes (SUVs) with encapsulated silibinin.

Samples	*ρ* [g/mL]	*γ* [mN/m]	*η* [mPa·s]
MLVs	0.939 ± 0.005 ^a,^*	28.7 ± 0.1 ^a^	3.45 ± 0.02 ^a^
UV-irradiated	0.917 ± 0.004 ^b^	27.1 ± 0.2 ^b^	3.28 ± 0.03 ^b^
SUVs	0.916 ± 0.006 ^b^	26.5 ± 0.2 ^c^	3.43 ± 0.02 ^a^

* Values with the same letter in each column showed no statistically significant difference (*p* > 0.05; n = 3; analysis of variance, Duncan’s *post hoc* test).

## Data Availability

The datasets generated during and/or analyzed during the current study are available from the corresponding author upon reasonable request.

## Supplementary Materials

The following supporting information can be downloaded at: https://www.mdpi.com/article/10.3390/pharmaceutics16111476/s1, Figure S1: FT-IR spectra of Phospholipon (Ph), Phospholipon after UV irradiation (Ph UV), empty liposomes, and empty liposomes after UV irradiation (empty liposome UV); Figure S2: The structure of phosphatidylcholine (a) and silibinin A (b); Figure S3: FT-IR spectra of silibinin, empty liposomes, silibinin-loaded liposomes (MLVs), and UV-treated liposomes for different periods of 15–90 min of UV irradiation in the 1550–1800 cm^−1^ spectral region; Figure S4: The deconvolution of the 1550–1800 cm^−1^ spectral region before and after 30 min of UV irradiation of Phospolipon (Ph) and empty liposomes; Figure S5: The deconvolution of the 1550–1800 cm^−1^ spectral region of the silibinin-loaded liposomes (MLVs) and their UV-irradiated samples after 15, 30, 45, and 90 min; Figure S6: The UV-initiated time-dependent change of the peaks in the 600–1300 cm^−1^ region for Phospolipon (Ph) and empty liposomes; Table S1: The results of deconvolution of the Phospholipon (Ph), empty liposomes, and silibinin-loaded liposomes (MLVs) before and after the defined period of UV irradiation; Figure S7: Raman spectra of the initial components Phospholipon (A) and silibinin (B); Figure S8: Effect of 24 h pre-incubation with the empty liposomes in a range of phospholipid concentrations (1, 10, 100, 250, 500, and 1000 µg/mL) on the cell viability of HaCaT cells *versus* control (represented by dashed line); determined by MTT assay. Data are expressed as mean + SEM relative to the unexposed control (dashed line); * *p* < 0.05 by one-way analysis of variance (ANOVA) with Tukey’s multiple comparison *post hoc* test; Figure S9: Effect of 24 h pre-incubation with the empty liposomes in a range of phospholipid concentrations (1, 10, 100, 250, 500, and 1000 µg/mL) on the production of reactive oxygen species in HaCaT cells *versus* control; determined by H2DCFDA assay. The data are expressed as mean + SEM; * *p* < 0.05 by one-way analysis of variance (ANOVA) with Tukey’s multiple comparison *post hoc* test; Figure S10: Effect of 24 h pre-incubation with the empty liposomes at a concentration of 10 µg/mL on the protein expression of cyclooxygenase-2 (COX-2), interleukin 1 beta (IL-1β), and macrophage inhibitory factor (MIF) in HaCaT cells *versus* control; using the cELISA method. The data are expressed as mean + SEM.
