# Supplementary material for: Silibinin-Loaded Liposomes: The Influence of Modifications on Physicochemical Characteristics, Stability, and Bioactivity Associated with Dermal Application
Source: Pharmaceutics. 2024 Nov 19;16(11):1476. doi: 10.3390/pharmaceutics16111476 (PMC11597119; doi:10.3390/pharmaceutics16111476)
Supplement: Supplementary file 1 [file pharmaceutics-16-01476-s001.zip › pharmaceutics-3304917-supplementary.pdf]

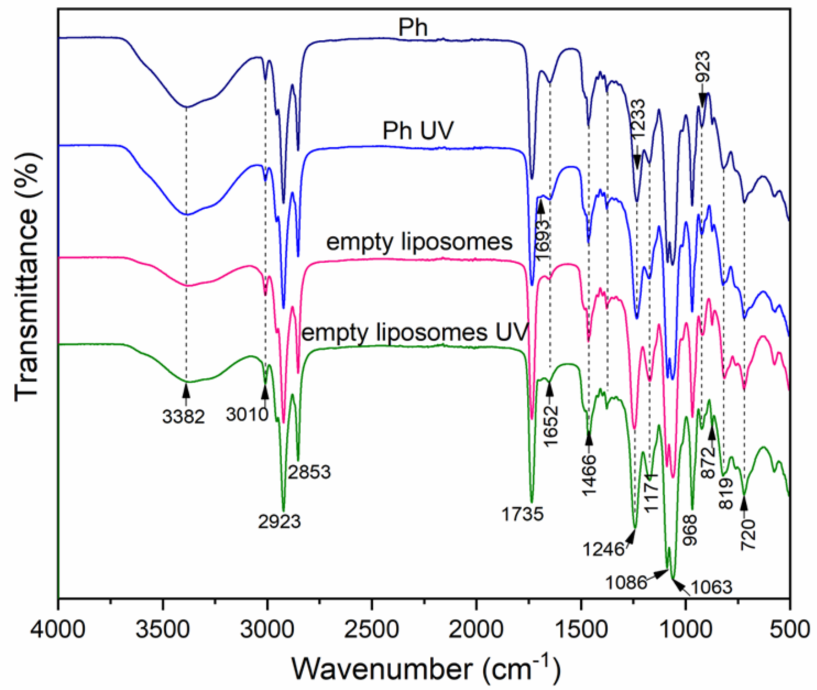

Figure S1. FT-IR spectra of Phospholipon (Ph), Phospholipon after UV irradiation (Ph UV), empty liposomes, and empty liposomes after UV irradiation (empty liposome UV)

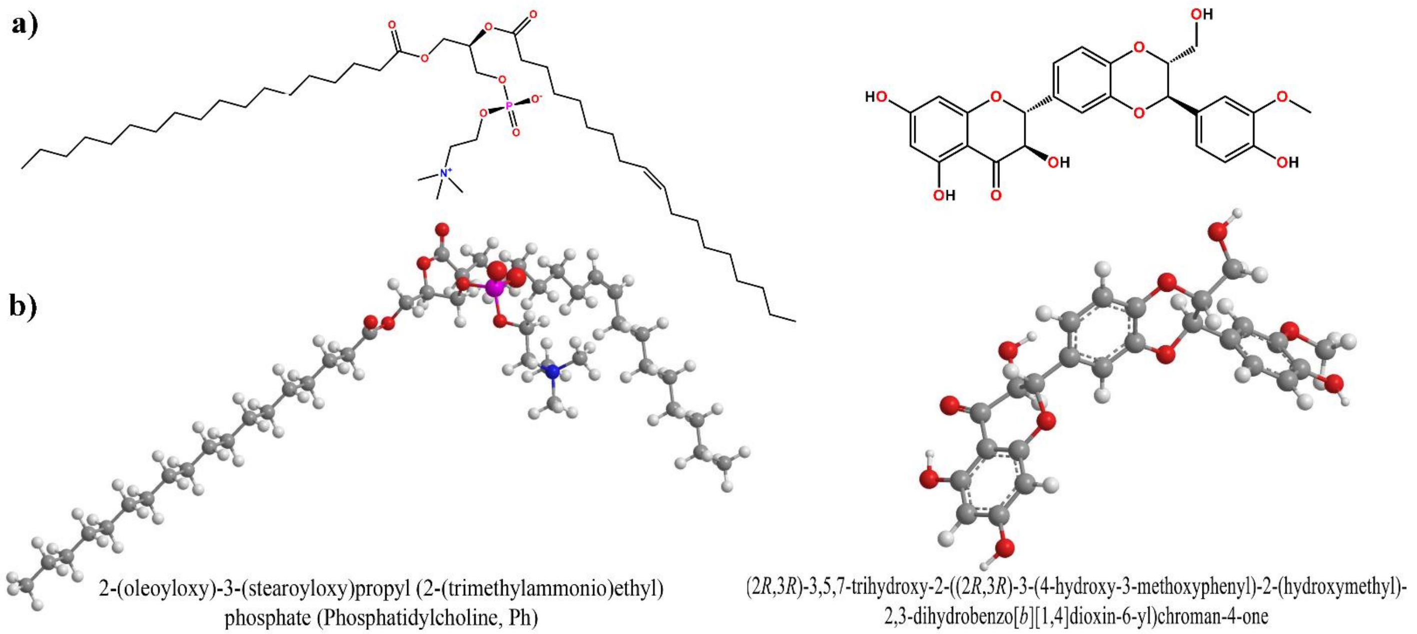

2-(oleoyloxy)-3-(stearoyloxy)propyl (2-(trimethylammonio)ethyl) phosphate (Phosphatidylcholine, Ph)

(2*R*,3*R*)-3,5,7-trihydroxy-2-((2*R*,3*R*)-3-(4-hydroxy-3-methoxyphenyl)-2-(hydroxymethyl)-2,3-dihydrobenzo[*b*][1,4]dioxin-6-yl)chroman-4-one (Silibinin A)

Figure S2. The structure of phosphatidylcholine (a) and silibinin A (b)

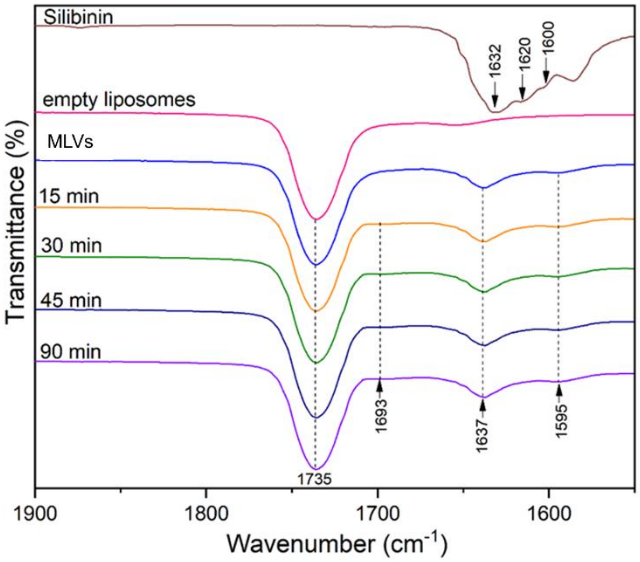

Figure S3. FT-IR spectra of silibinin, empty liposomes, silibinin-loaded liposomes (MLVs), and UV-treated liposomes for different periods of 15-90 min of UV irradiation in the 1550-1800 cm^{-1} spectral region

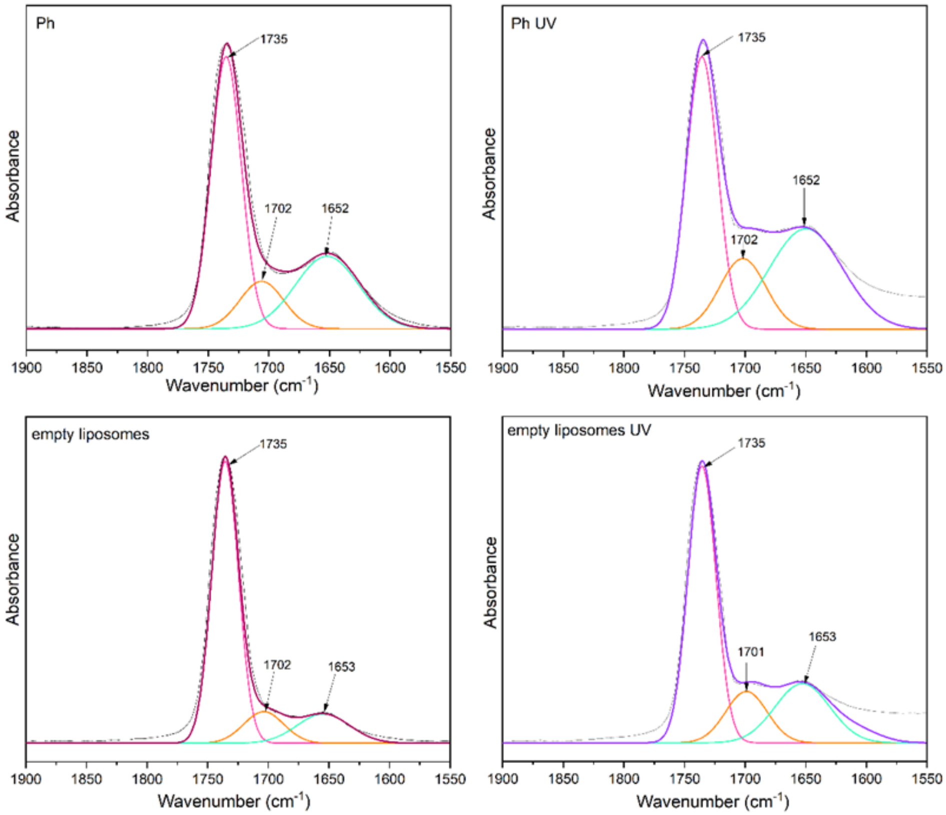

Figure S4. The deconvolution of the 1550-1800 cm^{-1} spectral region before and after 30 min of UV irradiation of Phospolipon (Ph) and empty liposomes

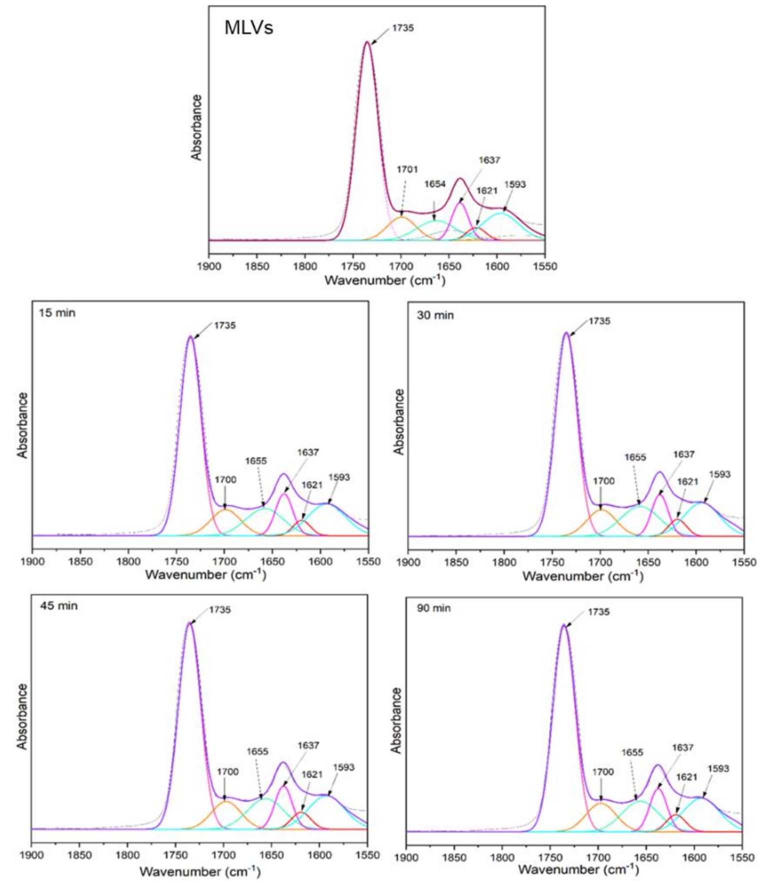

Figure S5. The deconvolution of the 1550-1800 cm^{-1} spectral region of the silibinin-loaded liposomes (MLVs) and their UV-irradiated samples after 15, 30, 45, and 90 min

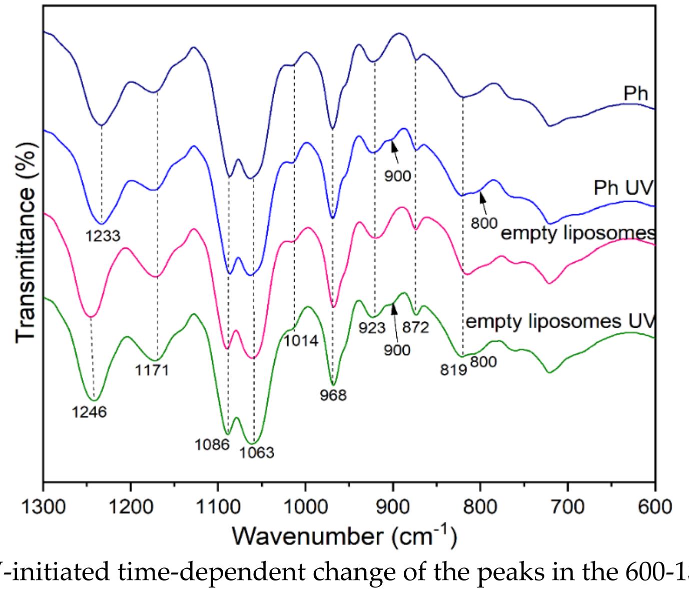

Figure S6. The UV-initiated time-dependent change of the peaks in the 600-1300 cm^{-1} region for Phospolipon (Ph) and empty liposomes

Table S1. The results of deconvolution of the Phospholipon (Ph), empty liposomes, and silibinin-loaded liposomes (MLVs) before and after the defined period of UV irradiation

Samples	Deconvoluted peaks	X (cm^{-1})	Area (cm^2)	%
Ph	Peak 1	1735	791.67	55.3
	Peak 2	1702	198.82	13.9
	Peak 3	1652	440.40	30.8
Ph UV	Peak 1	1735	812.43	44.4
	Peak 2	1702	310.76	17.0
	Peak 3	1652	706.46	38.6
empty liposomes	Peak 1	1735	829.91	72.7
	Peak 2	1701	139.62	12.2
	Peak 3	1653	171.32	15.0
empty liposomes UV	Peak 1	1735	861.39	59.1
	Peak 2	1701	235.86	16.2
	Peak 3	1653	360.87	24.7
MLVs	Peak 1	1735	788.89	56.3
	Peak 2	1701	126.57	9.0
	Peak 3	1654	140.64	10.0
	Peak 4	1637	110.64	7.9
	Peak 5	1621	40.17	2.9
	Peak 6	1593	193.50	13.8
15 min	Peak 1	1735	811.94	51.7
	Peak 2	1700	155.54	9.9
	Peak 3	1655	181.86	11.6
	Peak 4	1637	140.38	8.9
	Peak 5	1621	51.40	3.3
	Peak 6	1593	230.21	14.5
30 min	Peak 1	1735	854.54	50.3
	Peak 2	1700	174.56	10.3
	Peak 3	1655	202.41	11.9
	Peak 4	1637	155.85	9.2
	Peak 5	1621	60.45	3.6
	Peak 6	1593	250.24	14.7
45 min	Peak 1	1735	888.94	49.0
	Peak 2	1700	185.90	10.4
	Peak 3	1655	215.41	12.1
	Peak 4	1637	165.76	9.3
	Peak 5	1621	63.36	3.5
	Peak 6	1593	263.99	14.8
90 min	Peak 1	1735	888.99	49.8
	Peak 2	1700	186.01	10.4
	Peak 3	1655	217.52	12.1
	Peak 4	1637	166.16	9.3
	Peak 5	1621	63.41	3.5
	Peak 6	1593	264.00	14.8

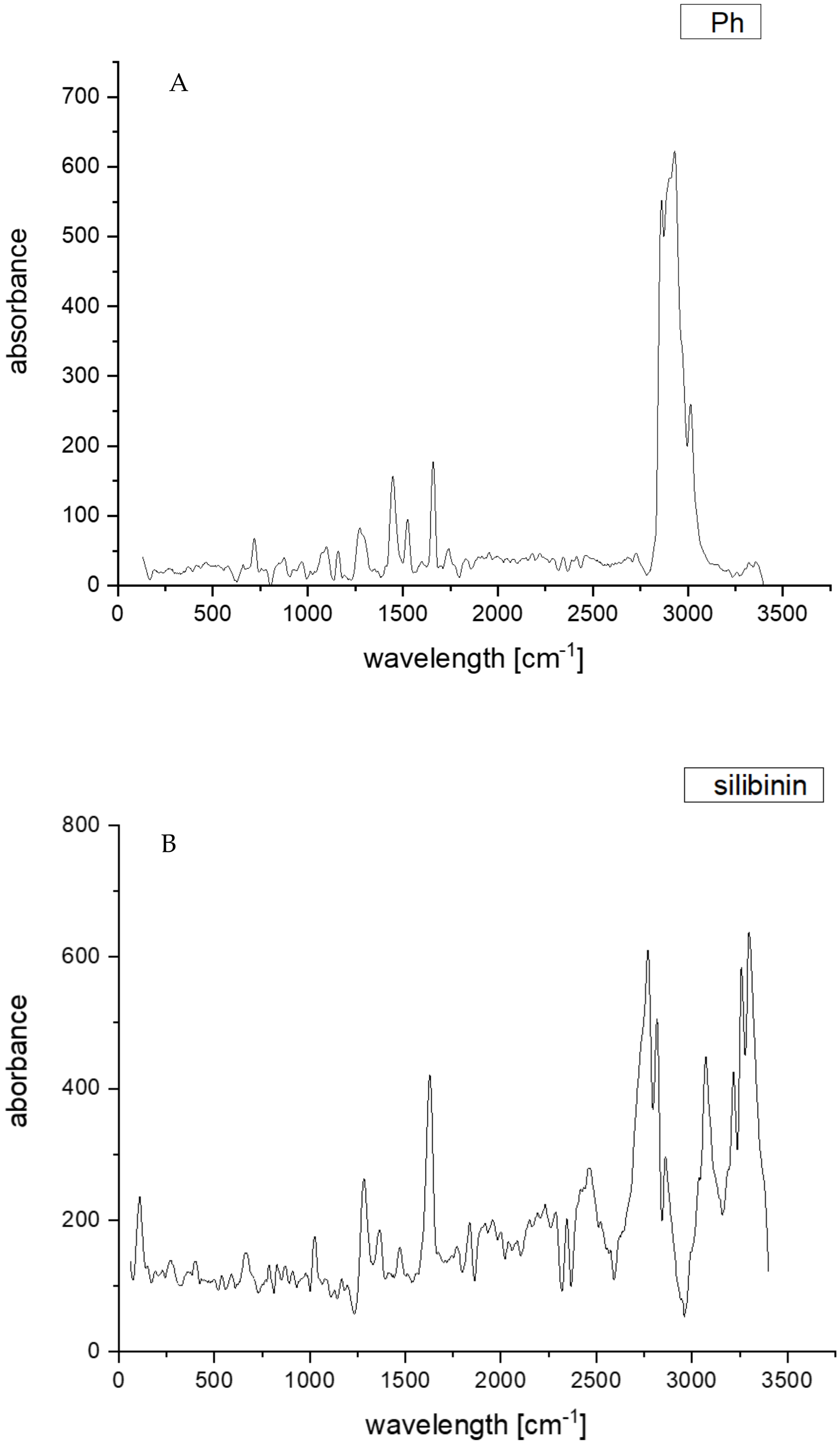

Figure S7. Raman spectra of the initial components Phospholipon (A) and silibinin (B)

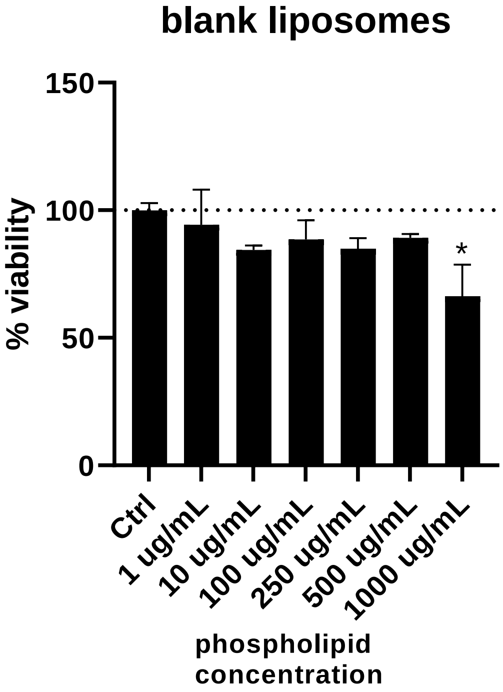

Figure S8. Effect of 24 h pre-incubation with the empty liposomes in a range of phospholipid concentrations (1, 10, 100, 250, 500, and 1000 μg/mL) on the cell viability of HaCaT cells *versus* control (represented by dashed line); determined by MTT assay. Data are expressed as mean+SEM relative to the unexposed control (dashed line); * $p<0.05$ by one-way analysis of variance (ANOVA) with Tukey's multiple comparison *post hoc* test

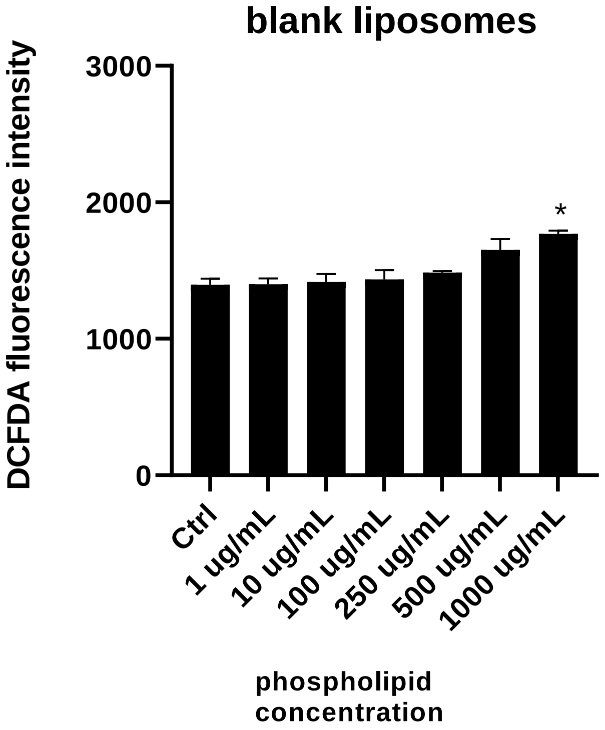

Figure S9. Effect of 24 h pre-incubation with the empty liposomes in a range of phospholipid concentrations (1, 10, 100, 250, 500, and 1000 μg/mL) on the production of reactive oxygen species in HaCaT cells *versus* control; determined by H2DCFDA assay. The data are expressed as mean+SEM; * $p<0.05$ by one-way analysis of variance (ANOVA) with Tukey's multiple comparison *post hoc* test

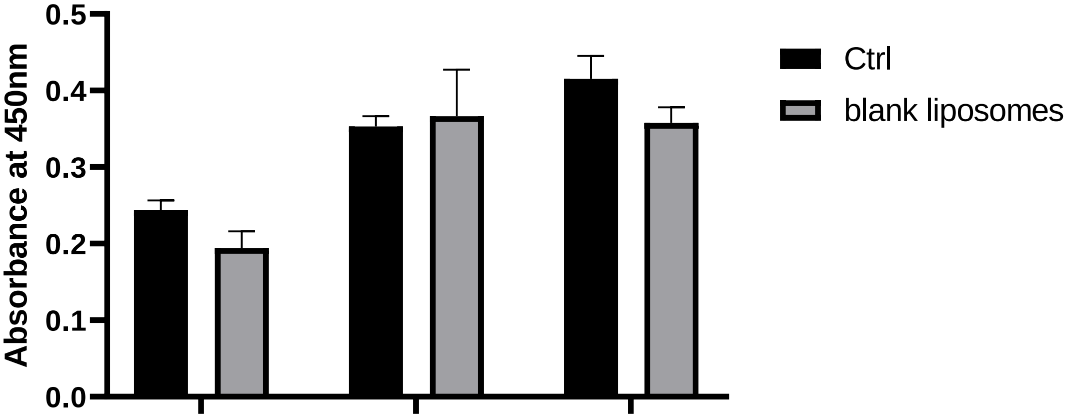

Figure S10. Effect of 24 h pre-incubation with the empty liposomes at a concentration of 10 μg/mL on the protein expression of cyclooxygenase-2 (COX-2), interleukin 1 beta (IL-1β) and macrophage inhibitory factor (MIF) in HaCaT cells *versus* control; using the cELISA method. The data are expressed as mean+SEM
